# Comprehensive security review and challenges for smart and power grids to prevent cyber-attacks in IoTs

**DOI:** 10.1016/j.mex.2026.104043

**Published:** 2026-07-13

**Authors:** Maha Kadhim Kabier, Zaid Ameen Abduljabbar, Mohammed Abdulridha Hussain, Vincent Omollo Nyangaresi, Zahraa Abdullah Ali, Hamid Ali Abed AL-Asadi, Abdulla J.Y. Aldarwish, Ali Hasan Ali, Ali A. Yassin

**Affiliations:** a Department of Computer Science, College of Education for Pure Sciences, University of Basrah, Basrah, 61004, Iraq; b Ministry of Education, General Directorate of Education in Maysan, Maysan, 62001, Iraq; c Shenzhen Institute, Huazhong University of Science and Technology, Shenzhen, 518000, China; d Department of Computer Science and Software Engineering, Jaramogi Oginga Odinga University of Science and Technology, Bondo, 40601, Kenya; e Department of Applied Electronics, Saveetha School of Engineering, SIMATS, Chennai, Tamil Nadu, 602105, India; f Department of Cybersecurity Science, College of Science, ALkunooze University, Basrah, 61004, Iraq; g Department of Mathematics, College of Education for Pure Sciences, University of Basrah, Basrah, 61004, Iraq; h Technical Engineering College, Al-Ayen University, Thi-Qar, 64001, Iraq; i Institute of Mathematics, University of Debrecen, Pf. 400, Debrecen, H-4002, Hungary

**Keywords:** Authentication, Smart grid, Power grid, Cyber threats, IoTs

## Abstract

One of the most significant use cases of the Internet of Things (IoTs) is the Smart Grid (SG), which is an advanced digital bi-directional power distribution system characterized by self-recovery capabilities. This SG offers resilient, adaptive, self-recovery, and sustainable power distribution. The recent past has seen a gradual shift from unidirectional power systems towards the SG’s bi-directional model. However, these large, complex, and self-healing SG systems have numerous vulnerabilities that threaten the exchanged data and power control commands. Therefore, many research efforts have been directed towards the enhancement of the SG security. In this paper, an overview of threats, attacks, vulnerabilities, security measures, and limitations for the IoT-based SG are described. We also give detailed descriptions of the Supervisory Control and Data Acquisition (SCADA) architecture as well as cloud computing as critical components of the smart grids. Moreover, this survey paper discusses tools and methods for verifying security protocols. The outcomes indicate the necessity of ensuring Quality of Service (QoS) in various communication technologies and networks deployed in SGs. The findings also point to the significance of developing network models and middleware to enhance QoS of the smart grid networks, which collect various data from diverse smart components and sensors.

## Review Highlights

In this paper we make the following contributions:.

• We provide an overview of threats, attacks, vulnerabilities, security measures, and limitations of IoT-based smart grid.
• Detailed descriptions of the critical components of the smart grids such as SCADA architecture and cloud computing are given.
• We present detailed analysis of tools and methods for security protocols verification.

## Graphical abstract

**Figure:**
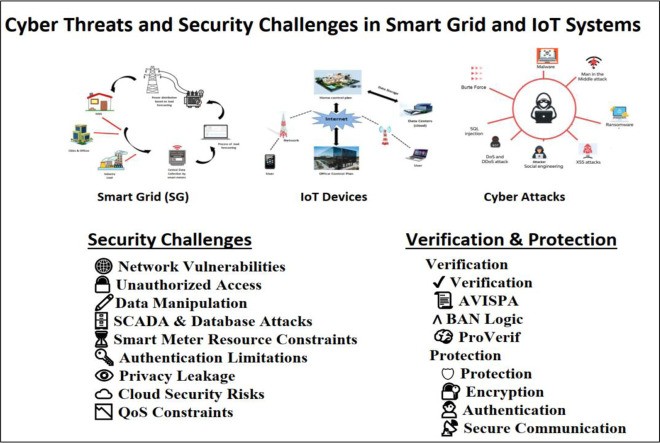

**Specifications table**

**Table:** 

**Subject area**	Computer Science
**More specific subject area**	Cybersecurity in Smart Grids and Internet of Thing
**Name of the reviewed methodology**	Thematic literature review
**Keywords**	Authentication, smart grid, power grid, Cyber threats, IoTs
**Resource availability**	Not applicable
**Review question**	What are the most critical security threats, vulnerabilities and attacks in the smart grid ecosystem? Which are some of the effective security measures that can protect IoT-based smart grid? What are limitations of the conventional countermeasures deployed for security protection in the smart grid environment? How are SCADA and cloud computing architectures designed to support smart grid operations? Which are the most popular tools and methods for security protocols verification?

## Methodological foundation and analytical framework

Using a thematic literature review and analysis framework, this study examines the facets of SG/IoT/AMI/SCADA-based environments in terms of cybersecurity aspects, including communication protocols, attack models, authentication processes and defense mechanisms. The methodology of this review was designed to leave no stone unturned, extract relevant findings and systematically map the existing evidence on smart grid cybersecurity and IoT-enabled infrastructures. Our work phases are: literature collection and source identification, thematic classification and research organization, cybersecurity threat and attack analysis, comparative Analysis of communication protocols, crash course in security threats (new ones added every day), flag critical review of existing countermeasures, as follows:

## Phase 1: literature and standards review

The primary phase involved collecting applicable articles from huge scientific databases, including Elsevier ScienceDirect, SpringerLink, IEEE Xplore, Wiley Online Library and MDPI journals. Other references have been collected from papers and reports, standards, RFC specifications and cybersecurity recommendations for smart grid and IoT communications systems. To cover both basic and contemporary work on smart grid cybersecurity, the analysis papers were primarily published from 2010 to 2025.

## Background

The traditional electric Power Grids (PGs) infrastructure utilized in many power companies is outdated and hence is incapable of taking advantage of the rapid developments in modern Information and Communication Technologies (ICTs). To address these concerns, the SG networks have been developed. These networks are based on recent developments in technology and communications. SGs provide better services during the transmission and distribution of electric power, as well as real-time tracking of energy consumption [1,2]. The implementation of smart grids necessitates the widespread use of smart devices, such as sensors, actuators, Smart Meters (SMs), and embedded computers. These smart devices gather data in real-time about electricity consumption patterns as well as the conditions of distributed energy resources and other grid components [3]. However, the high volume of diverse data collected by smart grid components for measurement and monitoring in these smart networks needs to be exchanged in a trustworthy and secure manner [4,5].

The way energy is controlled has changed as a result of the PGs increasing sensing and monitoring capabilities occasioned by the inclusion of SGs in power systems. For instance, the SMs can track power consumption over time and send relevant automated reports to the network operator. As such, smart meters give the power controller and the client relevant information that they may deploy for decision-making. For instance, the consumers/clients can use this data to change their behavior, devoid of the need for manual meter readings. Due to the SG-enabled real-time monitoring, power providers can easily detect faults in the local distribution network as soon as they arise, instead of depending on consumers to report outages [6]. According to [7], smart energy grids utilize a large number of devices throughout the transmission and distribution network, such as smart meters. This helps in offering two-way connections that monitor power consumption and supply.

Another benefit of smart grids is that smart meters can be utilized to combat the issue of electricity theft. In addition, solar power plants may be monitored and optimized for maximum solar energy harvesting courtesy of smart meters [8]. This is normally achieved through dynamic adjustment of the angle of the panels [9]. However, smart grids have many security challenges due to the public links being deployed for communication between the energy systems and other smart devices. In addition, the advanced devices used in SGs are resource-constrained and heterogeneous. This renders the provision of robust security using sophisticated cryptographic approaches quite challenging. As such, the SG ecosystem has continued to be vulnerable to a large number of security attacks whose sophistication and frequency keep increasing day by day [10]. Some of the notable attacks in smart grids include Man in the Middle (MITM), Denial of Service (DoS), and Distributed Denial of Service (DDoS) [9], physical capture, impersonation [11], replay, forgery, eavesdropping, spoofing, insider, Ephemeral Secret Leakage (ESL), privileged insider, guessing, desynchronization, structured query language injection, brute force, and repudiation. All these attacks call for an effective and efficient security system to mitigate them and preserve the security, privacy, and integrity of data [12]. Due to the criticality of SG devices, detection and prevention of attacks and tampering should be proactive. This will facilitate the generation of accurate notifications when any bug or problem occurs.

To mitigate the above threats, several different schemes have been proposed over the recent past. Many of these schemes focus on data protection, as well as integrity and confidentiality preservation [12]. Since cyber threats are dynamic in nature, staying ahead of adversaries requires proactive actions and ongoing enhancement of security mechanisms. To effectively address these issues and safeguard smart grids against possible security breaches, a multifaceted strategy is needed. This may encompass technical fixes, regulatory frameworks, stakeholder cooperation as well as ongoing research and development [13]. In addition, several security measures, including encryption, authentication, intrusion detection systems, and secure communication protocols, are essential for the protection of the SG infrastructure from cyber-attacks [14]. The primary contributions of this study are summarized as follows:

• We present detailed background on power grids and smart grids based on IoTs and other information communication technology components.
• Elaborate description of the most famous attacks on the smart grids is provided, including their effect on the electric power networks.
• To mitigate these security threats and attacks, we describe numerous proposed solutions and approaches.
• We underpin the significance of the common session keys, as well as the importance of efficient authentication approaches.
• We exhaustively examine the various types of communication protocols used in IoT-based smart grid networks.

**Fig. 1:**
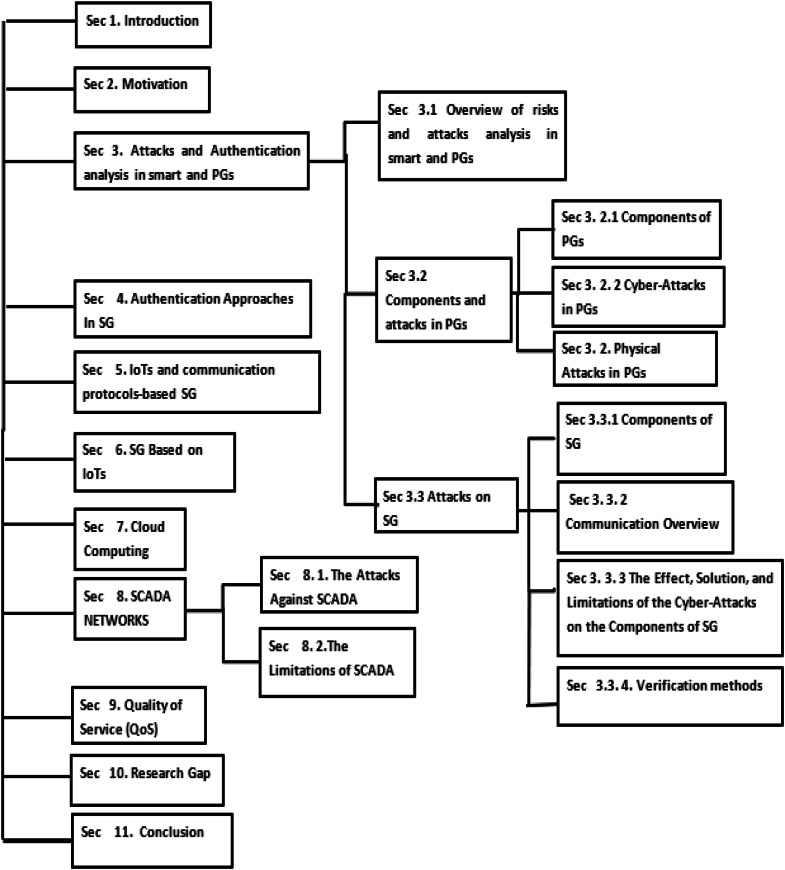
The organization of the paper.

**Fig. 2:**
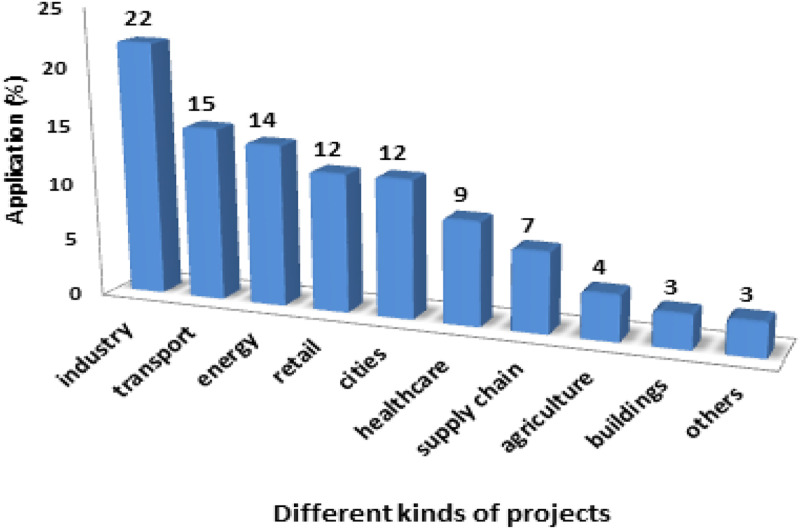
IoTs applications for different global projects [15].

The paper is organized as follows: Section 2 discusses the motivation behind this study, while Section 3 describes the various components of PGs and SG, as well as the attacks against these components. On the other hand, Section 4 investigates the various authentication approaches in SG, while Section 5 discusses IoTs and communication protocols applicable in smart grids. In Sections 6, 7, 8 and 9, SG based on IoTs, Cloud-based SG, SCADA networks and the attacks they are exposed to, and QoS issues in SG are discussed. Finally Section 10 discusses the emanating research gaps in this research domain. The organization of the paper is summarized in Fig. 1, while the various global projects deploying IoT are presented in Fig. 2.

## Motivation

Cryptography is highly significant in the preservation of data confidentiality, integrity, and authenticity within cyber-physical systems. Particularly, it has significantly contributed in the protection of personally identifiable information as well as control data. However, any successful compromise of the deployed cryptographic keys renders the entire system insecure [16]. Therefore, securing the cryptographic keys used in data encryption and decryption is crucial as this determines the integrity of any encryption system. In smart grids, there is frequent collection of data. Since this data contain details such as individual appliances and the times they were utilized, it is regarded as being extremely sensitive. In spite of this, the SG system uses the open Internet as the communication channel for the transmission of data generated by numerous smart devices. As such, there is a significant risk of information leakage or capture by different kinds of attackers [17]. This might lead to security threats such as disruption between supply and demand, impaired functionality of the smart devices among other security threats. In addition, insider attacks on the data collected by different smart devices may result in the improper management of different forecasting models, which are intended to balance demand response management with user satisfaction [18]. Therefore, it is imperative to mitigate or minimize as these vulnerabilities and attacks so that smart grid processes related to demand response and data analytics are correctly executed.

### Adapting conventional security architectures to modern smart grid environments

This study provides an extensive review of the gaps in existing authentication and communication mechanisms in current smart grid and Internet of Things (IoT) environments, aiding the adaptation of legacy and traditional security protocols in industrial applications. Many legacy technologies in SaaS platforms, industrial control systems and product development were originally designed for centralized, resource-rich infrastructures and are therefore less suited to the distributed, heterogeneous and resource-constrained environments of today. The research provides the security vulnerabilities and performance bottlenecks of conventional approaches. It also focuses on lightweight cryptographic algorithms, key exchange protocols and authentication frameworks that can be integrated into systems with minimal architectural changes to the original system. The study exposes the compatibility with established frameworks like TLS, hash-based authentication and ECC-based communication models, which would allow organizations to improve security gradually without moving away completely from their legacy systems. This paper provides a systematic review of existing data on current threats, attack models and mitigation techniques and offers guidance to switch conventional systems to adaptable security architectures that can evolve with current technology advances in Smart Grid, IoT, SaaS and industrial environments by being scalable, resilient and adaptive.

### Study selection procedure and filtering criteria

The search utilized a variety of keyword sets categorized for smart grid and IoT security, SCADA systems, Advanced Metering Infrastructure (AMI), lightweight authentication, malware injection, ransomware and communication protocols. In this study, a multi-stage screening procedure was used for systematic screening following a transparent, uniform and repeatable process. The associated publications were extracted from major scientific databases such as IEEE Xplore, ScienceDirect, SpringerLink, Wiley Online Library and MDPI Google Scholar. Research was selected over a series of four sequential steps. Automatic detection and then manual validation and removal of the duplicated records retrieved from different databases were the first step. Then, we carried out title and abstract filtering to filter out non-related works to smart grids, IoT-enabled power systems, industrial control systems, or cybersecurity applications. We conducted a depth assessment to study the methodological rigour and technical relevance of each article for full-text eligibility. The rest of the papers were then clustered within paper thematic groups, as well as with the major sections of this review.

We searched all peer-reviewed journal papers, conference papers, industry standards, RFC specifications, and technical reports, including highly cited literature on smart grid security and communication protocols, and covering cyber-physical attacks, as well as numerous aspects such as authentication mechanisms and IoT integration. The exclusion criteria were duplicate publications, studies unrelated to a dimension of the smart grid domain, papers lacking technical details, non-English publications, inaccessible full-text papers, and low-weight articles in terms of cybersecurity or communication infrastructure. To maintain analytical homogeneity, the filtered studies were selected based on their publication quality, citation impact, publication recency, experimental validation, and utility for practical deployment. We focused on solutions that were lightweight, easy to scale and deploy for large-scale smart grids, as well as those suitable for resource-constrained IoT deployments.

## Phase 2: thematic classification and research organization

First, the literature collected during the systematic mapping process was classified into one or more research themes using a framework developed in the paper. The various categories of studies in the analyses included smart grid architectures, IoT integration, communication protocols, SCADA systems, authentication schemes and cyberattack models (including ransomware threats, False data injection attacks and desynchronization attacks), lightweight cryptographic mechanisms and key management strategies. Thematic organization helped provide a more logical flow of analysis between the various questions posed and the cybersecurity challenges and defence strategies articulated in this article.

### Attacks and authentication analysis in smart and PGs

Conventional power grids are central systems in which the energy flow is unidirectional through transmission and distribution lines. These conventional unidirectional data flow has been demonstrated to be problematic. As such, smart PGs have been developed to provide a two-way communications infrastructure for the generation and consumption of distributed energy in a [19].

#### Overview of risks and attacks analysis in smart and PGs

Security threats in smart grids include virus propagation, malfunctions of cyber system, adversaries gaining access to private customer information, as well as vulnerabilities in distributed control devices [20]. The traditional power grids face numerous challenges such as chronic blackouts, energy storage issues, asset costs, and high carbon emissions [21]. To address some of these issues, smart grids have been developed to provide reliable, efficient, and clean energy distribution. Unfortunately, smart grids are vulnerable to cyber-attacks that can compromise security requirements [2,[22], [23], [24], [25]]. In the following sub-sections, overview of PG and SG attacks are described.

#### Distinction between smart grid threat models and software CVEs

The critical threats that this study covers are distinct from the lower-level modern industry Common Vulnerabilities and Exposures (CVEs), though they are certainly more intertwined in specific contexts. Common Vulnerabilities and Exposures (CVEs) are defined, well-documented implementation-level vulnerabilities found in specific software versions due to coding errors or misconfigurations, or a lack of input validation, such as those in popular applications like Apache Log4j and Oracle products, or in the Linux kernel. This work investigates risks (man-in-the-middle, replay, impersonation, and denial-of-service) that fall into universal attack categories or adversarial capabilities independent of a specific implementation in smart grid and IoT communication models. Where the CVE is an instance of an attack class, only then can these be viewed as being equivalent. The infamous Log4j vulnerability (Log4Shell) can enable remote code execution, which could further escalate attacks through data manipulation, impersonation, or denial-of-service threats consistent with those reported in smart grid-specific security reviews. Likewise, CVEs in operating systems or middleware (e.g., Linux or Oracle systems) can be exploited to execute denial-of-service or privilege escalation attacks that align with the abstract risks delineated in this study. However, they differ in context: CVEs are implementation-specific and can be patched, while the dangers explored in this work are systemic and apply at the protocol level, requiring cryptographic design, secure authentication and formal verification for mitigation. The work here is at a different level of abstraction. The aim is to design protocols that remain secure even when vulnerabilities exist at lower levels, rather than patching specific CVEs.

#### Components and attacks in PGs

1. Components of PGs

• **Generation**: Power plants generate electricity from different sources such as natural gas, coal, nuclear, solar, hydro, and wind. Whereas thermal power plants use heat to generate electricity, hydroelectric plants generate power by harnessing the energy produced by descending water. On the other hand, renewable energy sources such as solar and wind are deployed by power plants to produce electricity [26].
• **Transmission**: Electric power transmission networks transport electricity from power generation plants to substations. Therefore, this component is characterized by high-voltage power lines with a large transmission line capacity. Basically, the substations are dispersed across the transmission network and are used to adjust the electric voltage [26].
• **Distribution**: Distribution networks carry electricity from substations to customers. Basically, distribution lines are lower-voltage power lines that carry electricity to neighborhoods and businesses. Here, transformers are used to reduce the electrical voltage to a level suitable for safe use in homes and businesses [26].
• **Customer use**: Customers utilize electricity for a variety of purposes, such as lighting, heating, cooling, and appliances. At the customer premises, electricity is typically measured in kilowatt-hours (kWh).
• **Energy consumption data**: This is data collected regarding the quantity of energy consumed by people, homes, companies, or entire nations. It contains information on the amount of gas, electricity, and other energy used over a given time frame. Basically, this data relates to the power usage of different service systems and their components. Such data is influenced by factors such as loads for heating and cooling, natural gas usage among others [27].

With the rapid growth and adoption of the IoTs in power systems, these components are inherently heterogeneous. In addition, typical PGs comprise of a wide range of networks, as well as various states such as generation, transmission and consumption. These issues, coupled with the long transmission distances expose PGs to attacks. These security threats can have high impacts as well as low frequency disturbances that cause equipment damage, loss of load, and instability of the power systems [28].

2. Cyber-attacks in PGs

The power grids are increasingly connected to the Internet, which is an open and public communication channel. This renders these grids vulnerable to cyber threats. Some of the common cyber attacks in the environment are described below.

• **Malware:** This attack involves the introduction of malicious peace of code into PGs systems. The effects may include the compromise of grid operations. In addition, these malware can allow attackers to gain access and control of the power systems components [27].
• **Distributed Denial of Service (DDoS):** This attack involves the flooding of the PGs network with massive amounts of traffic. The effects include rendering the power grids inaccessible, as well as the disruption of electricity delivery [29,30]**.**
• **Phishing:** in this security threat, adversaries trick employees into providing confidential information through emails or other means. This confidential information can be used to gain unauthorized access to PGs infrastructure [27].
• **Denial of Service (DoS):** The main aim of this attack is to render the Remote Terminal Unit (RTU) unavailable for the controlling station. Basically, the adversary bombards one of the power grid entities with a large volume of duplicate requests, making it impossible for that entity to handle them. This can effectively incapacitate this component to an extent that it cannot respond to legitimate requests delivered to it [31].

3. Physical attacks in PGs

Apart from the cyber-attacks discussed above, power networks are also vulnerable to physical attacks that directly damage their infrastructure. The most common physical attacks [32] are described below.

• **Vandalism:** These are intentional acts of destruction, such as cutting power lines, damaging substations, or destroying transformers, that can disrupt the flow of electricity.
• **Theft:** This refers to the stealing of critical equipment such as copper wires or transmission components, which can affect network functions and cause power outages.
• **Terrorist attacks:** This refers to terrorist organizations targeting power generation systems as a means of creating widespread chaos and disruption [32]**.**
• **Conditions Facilitating Covert False Data Injection Attacks:** Specifically, when the injected measurements are engineered to mathematically match the physical and operational model of the power system, a class of cyber attacks called False Data Injection (FDI) can evade conventional bad-data detection, state estimation and control center validation schemes. Typically, smart grid state estimation uses residual analysis as a basis for detecting bad data, along with statistical thresholds and measurement-consistency assessment. The condition is that the attacker knows enough about the grid topology, the measurement matrix, how measurements are communicated, or the operational conditions to create synchronised incorrect measurements that are consistent with a basic set of state estimation equations and still modify the estimate of the system state. Under such conditions, the telemetry modification yields residual values below the acceptable detection limits; as a result, the fake data appears genuine to the control center. It makes such attacks possible when adversaries gain simultaneous access to multiple measurement points, compromise communication channels between the RTUs, smart meters, PMUs (Phasor Measurement Units) and SCADA components, or, most often, exploit the lack of effective authentication and integrity protection mechanisms in legacy industrial protocols. The sparsity of sensor deployment, imperfect observability, delayed synchronization, and insufficient redundancy hinders the bad-data detection techniques. Advanced adversaries can use known load profiles, historical Operational Data, or Model-based machine learning to create covert injections that emulate true system behaviors. As a result, traditional techniques for validating telemetry may begin to fail when the difference between an intentionally coordinated telemetry manipulation event and operational changes in smart grid or distributed IoT-enabled environments shifts toward statistical anomaly detection.

In Table 1, more details regarding the types of attacks in power grids and their effects are presented. In addition, various mechanisms, actions, limitations, destructive effects, resolving methods, and prevention techniques are described in Table 1.

**Table 1:** Attacks types on power grid systems.

Attack type	Mechanism of attack	Effect of attack	Prevention method	Limitation of prevention method
Passive-Reconnaissance	Information-gathering techniques and listening, such as IP addresses [33].	Identify the device type, the Application Service Data Unit (ASDU) addresses, and the Information Object Addresses (IOA), the Object Addresses (OA), confidential information, including active commands or measured data values [33].	Zero trust includes, which includes establishing strict access controls based on user identity, encryption of data in transit, and protecting it from unauthorized access; Multi-factor authentication (MFA) [33].	It suffers from lack of untraceability mechanism [33].
Active- Reconnaissance	Mapping Remote Terminal Unit (RTU) pairing options by sending STARTDT (ASDU) messages with all IPs spoofed one by one in the subnet [33]. Address Resolution Protocol (ARP) poisoning for the controlling station and the RTU in order to gather information (MITM), flooding the switch.	Finding the RTU devices, controlling station, and the gateway to the protected network [33]. Locating the controlling station, RTU devices, and the secured network gateway.	Access control lists, intrusion detection systems, network segmentation, and encryption to protect against unauthorized scanning, probing, and information gathering activities. These measures can help mitigate the risk of successful active reconnaissance attacks [33].	Extra-large amount of traffic active-reconnaissance attacks can be time-consuming and resource-intensive [33].
Operation failure	Injecting packets to transmit false data on behalf of the RTU, or issuing unauthorized commands to open or close the breaker with ARP poisoning.	Sending wrong measurement values and trying to propagate the error.	Intrusion detection systems (IDS) and firewalls [33].	IDS does not prevent attacks, it only helps detect them. For this reason, IDS must be part of a comprehensive plan that includes other security measures.
Denial of Service	Flooding the ASDU RESET by means of packet injection combined with controlling station source IP spoofing [33]; Using packet injection and controlling station source IP spoofing, ASDU RESET flood.	Disable the communication channel between the RTU and the control station through the injection of spoofed RESET messages immediately following the reestablishment of the connection; disable RTU accessibility [33].	Load balancers, Firewalls, Intrusion detection systems (IDS) [33].	It has high computation cost [16].
CrashOverride	Hackers exploited the weakness of Microsoft Windows machines in its information technology (IT) and communication networks to access the system.	Control the on/off switching devices of the power transmission lines; It is not cataclysmic and would result in hours, potentially a few days, of outages for weeks or more [34].	There are currently no effective solutions that can fully address the threat posed by such attacks [33].	The functionality of the CRASHOVERRIDE framework serves no espionage purpose and is primarily designed for attacks that would lead to electric outages [34].
Malware attacks	Blackouts can cause widespread disruption, leading to lost productivity, financial losses, and even loss of life [27].	Widespread power outages, disrupt critical infrastructure, and affect public safety; stealing sensitive data; corrupting files, or taking control of a system [27].	Firewalls, intrusion detection systems, and regular system updates and patches [27].	High cost, some malware attacks are only effective against specific operating systems or software applications and thus requires special resistance systems [27].
Triton	Malware is designed to interact with Triconex Safety Instrumented System (SIS) controllers made by Schneider Electric and specifically the Triconex 3008 processor. The malware's intention is to disrupt the safety mechanisms of the controllers in the target facility [32]. Malware is specifically developed to interact with Schneider Electric Triconex SIS controllers, namely the Triconex 3008 processor. The goal of the malware is to interfere with the target facility's controllers' safety features.	Disrupt the safety mechanisms of the controllers in the target facility [33]; disrupt the target facility's controllers' safety mechanisms.	Firewalls, intrusion detection and prevention systems, and antivirus software; regularly updating and patching all software and hardware components [33].	The attack did not succeed in carrying out some active attacks such as active reconnaissance attacks due to the unresponsiveness of the RTU if the IP address did not match the pre-set IP address of the control station [35]**.**
VPNFilter	Once installed, VPNFilter attempts to connect to a Command and Control (C&C) server to receive instructions and download additional modules that extend the malware's capabilities. The malware uses obfuscation techniques to hide its communication with the C&C server, such as retrieving images from Photobucket and toknowall.com that contain the active URLs of the C&C server in their meta-data portion [32].	The devices' hard resets, patch applications, and default login configuration changes [36].	Keep software up to date, use a firewall, use an IDS, VPN; educate employees [36]**.**	It is not able to steal data that is encrypted at rest; it is not able to disable devices or networks completely; it needs a database to configure the filter; needs to update the database permanently and dynamically, which increases the volume of data in the base [37].
Black energy	A long-term reconnaissance of the system. It can hide in system for a long time, for months or even years, until it gets the knowledge it needs to attack the system or knowledge of system operation [38].	It can access internal network, and they can also deny service calls to report outages. In this way, the control center, which is far away from this substation, does not know what is happening in the remote substation.	Wirewalls, intrusion detection systems, and access control systems; monitor and update security systems; educate employees about security; use strong passwords and multi-factor authentication	It predominantly started as a botnet/DDoS tool. This indicates that while it has evolved and gained additional capabilities, its origins as a botnet/DDoS tool may impose limitations on its functionality and adaptability for more complex attack scenarios [32].
Injection attacks	The measurements are injected with false data in the SCADA communication network. This data is then received at the control center [39].	These false signals can lead to improper control actions being taken [40].	Validate all user input, use parameterized queries; use prepared statements; use a web application firewall; keep software up to date.	It has high complexity [33].

### Attacks on smart grids

#### Components of the smart grid

As already explained, the smart grid is an evolved form of the conventional electrical grid system that offers a more reliable and consistent electrical power supply. The key differences between the traditional and smart grid are extensively explained in Table 2. A smart power grid consists of a number of devices that are interconnected and heterogeneous. In its operation, the smart grid utilizes numerous digital devices for enhanced system observability and control. This necessitates the use of IoTs for reliable device integration and grid tracing [41,42]. The deployed devices are based on different standards and technologies that communicate through different protocols. This sheer number and heterogeneity of smart devices can result in a wide range of attacks towards the smart grid components. In the sub-sections below, descriptions of the various smart grid components, as well as the overview of the communication process are provided [43].

• **Smart Meter (SM):** This is the main component of the smart grid, which gathers sensitive data and sends the accumulated information to Utility Service Provider (USP) over open wireless channels [44]. It measures, logs, and provides power usage statistics to energy firms as well as consumers at hourly or more frequent intervals. Basically, the smart meter collects and sends real-time power consumption reports to the USPs. Therefore, it is the fundamental technology that offers sophisticated metering services that enable two-way communication between energy providers and consumers to enhance operations. However, the two key functionalities of the smart meter are dynamic pricing and demand-side management. It is normally installed in consumer buildings, presenting an embedded system that records a variety of electrical parameters for use in billing, price adjustments, situation awareness and other applications [45,46].
• **Registration Authority (RA):** In the context of the smart grid, the RA is a trusted entity that is responsible for registering and authenticating the smart grid devices. By ensuring that only authorized devices are able to communicate with each other, the RA plays a critical role in ensuring the security of the smart grid system [39].
• **Utility Service Provider (SP):** This is the company that offers services to utilities and electrical consumers [47].
• **The Control Center (CC):** This component manages user electricity consumption data, controlling power generation and distribution. In addition, it receives data from users after authentication and session key acquisition [41]. Customers can save energy and minimize costs by using the CC to manage their electrical demands and make relevant adjustments to their electricity consumption at any time [48].
• **Trusted Third Party (TTP):** In smart grids, it is assumed that the TTP is reliable. Its functions include the creation of system parameters. In addition, it is in charge of CC and smart meter registration [41].

1. Communication overview

**Table 2:** Conventional power grids Vs. smart grids.

Traditional power grids	Smart grids
*Centralized Control* In traditional grids, the management of electricity generation, distribution, and consumption is usually done by a small number of very large power plants.	*Decentralized Control* The use of decentralized control systems in SG makes it possible to employ more Distributed Energy Resources (DERs), including wind turbines and photovoltaic systems. Better power flow control is made possible by these systems.
*One way Communication* Power plants communicate mostly with consumers in one way fashion through traditional grids. Information flow between the grid's various components is restricted.	*Two-way communication* This made possible by smart grid components situated between a variety of elements, including customers. This makes it possible to monitor, control, and exchange data in real time. It also gives decision makers access to additional information.
*Limited automation* In conventional power grids, there is not much automation. It is a typical practice to monitor and control power systems manually. As such, it may take some time to react to errors or adjust to user demands.	*Sophisticated automation* The smart grid uses digital technologies such as sensors and smart meters along with sophisticated automation to instantly identify and react to problems or variations in demand.
*Demand stability* Conventional power systems are built to manage consistent and predictable power usages. As such, they find it difficult to control sporadic renewable energy sources and adjust to variations in demand.	*Integration of renewable energy* The smooth integration of renewable energy sources is a design feature of smart grid networks. They can use energy storage technologies and sophisticated forecasting to control the unpredictability of sources such as solar and wind.
*Infrastructure* In conventional power grids, the infrastructure is frequently less adaptive and flexible. As such, system modifications or upgrades can be expensive and time-consuming.	*Enhanced reliability and resilience* Smart grids can withstand disturbances and flexibility is a better manner. They can save downtime and increase overall reliability by swiftly locating and isolating defects.

The smart grid is characterized by the integration of renewable energy sources, Real-Time Pricing (RTP) for clients, Demand Response (DR) programs involving residential and commercial users, as well as quick outage detection [47]. Due to its enormous size and complexity, it is preferable to create a single Information Communications Technology (ICT) framework for the smart grid. Basically, the detailed picture of the entire communication network and how it integrates with the physical elements of the SG is provided by an ICT framework. This ICT component facilitates the power utilities' realization of the SG domain interoperability [47,49].

**Fig. 3:**
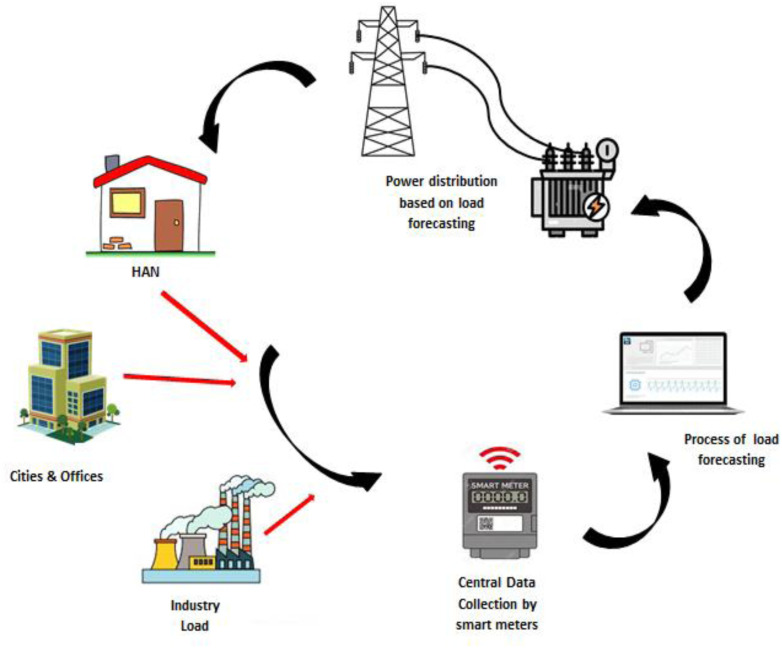
Data collection process in SG.

To monitor energy consumption in real time, smart homes operating in an SG environment have begun using smart meters. In order to achieve this, the smart meter and service provider exchange data over the open communication channels. As such, adversaries can alter, intercept, and interfere with the transmission process. This effectively lowers the smart grid system’s performance. Due to the numerous security threats associated with these channels, robust security and privacy are major requirements during the smart meter and service provider communications [50]. This can be attained by latest and sophisticated cryptographic techniques that cannot be easily compromised by attackers. However, the biggest challenge in the smart grids is the resource-limited nature of its devices in terms of memory, computation and communication. This is more pronounced on the side of devices such as smart meters [19]. Due to these limitations, a secure, lightweight, and reliable authentication protocol must be developed. This can help protect the privacy of network entities in addition to ensuring that information sharing is only amongst the legitimate members of the smart grid ecosystem [44]. As shown in Fig. 3, the smart meters are used to capture and send the collected data to the users and service providers. This gives consumers and the utility company easy and effective access to energy-related information [51]. The wireless communication among the smart grid components is achieved using numerous IoT approaches and technologies. These include Fifth Generation (5 G), Z-Wave, Low-power Wireless Personal Area Network (LowPAN), and ZigBee [42]. Owing to the continuous developments of various communication technologies, information technology in smart grid and IoT, there is increased diversity which serves to broaden the attack surfaces on smart energy networks.

2. Cyber-attacks in the smart grid ecosystem

Due to the large size and continued expansion of electric power networks, they have been extended to cover larger distances and deploy numerous diverse devices. As such, they have become inherently heterogeneous, linking with IoT technologies, as well as Renewable Energy Resources (RERs). These smart grids are crucial for reduction of environmental pollution as well as carbon dioxide emissions. However, smart energy networks face challenges such as their intermittent availability and heterogeneous technologies that increase risks and their vulnerabilities to attacks. In addition to the deployment of renewable energy sources, the smart grids infuse diverse data, including environmental state, storage status, and weather forecasts [47]. All this leads to the exposure of smart grids to many attacks that affect their functionalities or components. Some of the effects of these attacks are explained in this sub-section.

**Fig. 4:**
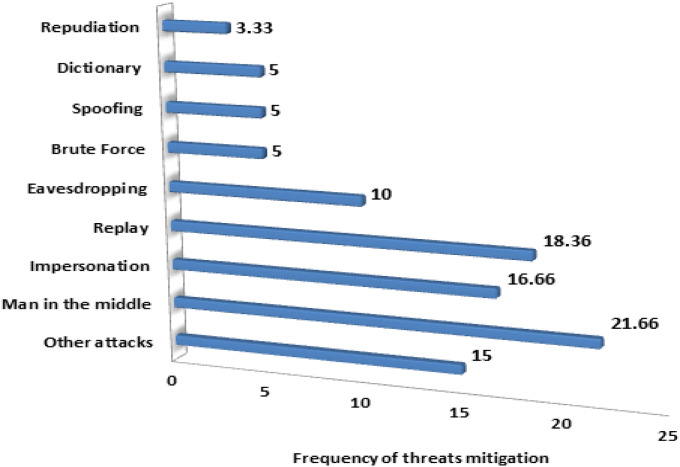
Frequency distribution of threat mitigation in SG [54].

Hackers can impersonate legitimate users, manipulate data, and perform cyber-physical attacks on IoT-based systems. Particularly, smart grid systems are vulnerable to DoS, MiTM, smart physical capture, DDoS, replay, forgery, impersonation, eavesdropping, spoofing, insider, Ephemeral Secret Leakage (ESL), privileged insider, guessing, desynchronization, structured query language injection, repudiation and brute force attacks. These attacks have serious repercussions that negatively affect the physical layer of the system, causing significant loss [52]. Privacy and confidentiality are other crucial aspects of the smart grid, as power monitoring systems can expose sensitive user information [53]. For instance, eavesdroppers can intrude into the smart energy networks and obtain critical user data. Most customers may not discard their receipts or bill properly, and this poses serious phishing risks. This can enable hackers to manipulate information, create fake messages or obtain crucial information [25]. Fig. 4 shows the frequency distribution of the mitigated cyber-attacks in smart grid.

• *Limitations of TLS in Smart Grid and IoT Environments*

The analysis views TLS as a de facto standard secure communication protocol and its effectiveness hinges on the successful incorporation of additional security measures (e.g., certificate validity, management of trusted CAs, key-exchange controls and defenses against possible downgrade attacks). The research mindset is to note that while TLS provides encryption, confidentiality and authentication at the transport layer, using it directly in smart grid and IoT settings will still be insufficient if implementation weaknesses or resource limitations are ignored. Most MITM write-ups that succeed in practice do not require an exploitable TLS state, but rather invalid certificate validation, poor trust management, a valid but expired and rogue certificate (i.e., a well-known public key), or improper protocol negotiation.

Thus, the research highlights an urgent need to blend transport layer security with weak mutual authentication, sound key management techniques, secure session establishment schemes and protocol verification methods for resource-constrained smart grid infrastructures. It seems for this reason that TLS is called a minimal security reference model and not regarded as an all in one solution. The rationale for the research is to investigate how to improve traditional secure communication mechanisms to adapt them to the operational and security constraints of smart grids and IoT systems.

The following are the most important attacks on the SG:

• *Man-In-The-Middle (MITM)*

**Fig. 5:**
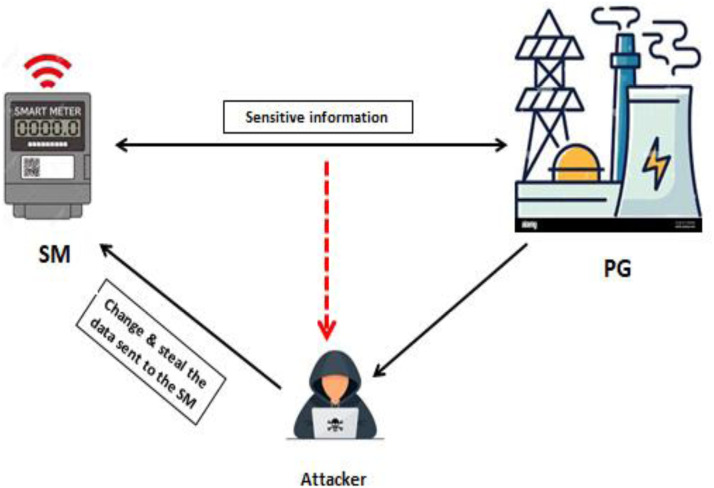
MITM cyber-attack in smart grid.

**Mechanism:** In a smart grid, MITM occurs when a malicious actor places himself/herself in the path of two authorized devices. He/she can then establish a connection with them, and relay the communication between them. The aim of the attacker is to steal the sensitive information passed across the open communication lines in the smart grid [52]. The impact of MiTM in smart grid is unauthorized access to sensitive information [2]. The first victim system is targeted when the attacker sends signals to it claiming to be the second victim system. At the same time, the attacker simultaneously sends different signals to the second victim system stating that it is the first victim system. Through this attack, the first victim sends the data to the second victim through the attacker. Upon establishing the illegitimate connection, the victim believes they are utilizing their regular network connection. The most popular method for executing MitM attacks is to alter the Media Access Control (MAC) address information through Address Resolution Protocol (ARP) poisoning, which is the process of exploiting the ARP weaknesses [55]. Fig. 5 presents a typical MitM cyber-attack in smart grids.

**The effect on the smart grid:** Attackers can use a MiTM attack to capture sensitive data sent to or from the smart meter, allowing them to monitor energy usage or steal sensitive data. In addition, attackers can deploy MiTM attacks to change and steal the data sent to the smart meter. This could effectively disable the smart meter or change how it works. In TTP, data privacy and integrity are compromised by intercepting and potentially altering traffic between two hosts. As such, this attack can also facilitate the breach of confidentiality and integrity [41]. In a nutshell, MiTM threats can potentially avert transmission of network data, alter it during transit and gain unauthorized access to valuable information. At the same time, these attacks can compromise network confidentiality and integrity [2]. For instance, adversaries can steal the data transferred between the data concentrator device and smart meter [56].

**Solution and limitation:** Transport Layer Security (TLS) is crucial in MiTM attacks prevention. Basically, the operation of TLS encompasses the encryption and securing of transactions through the exchange of keys prior to the execution of transactions. As such, TLS facilitate secure communication among various network entities across the internet [57]. Apart from TLS, the use of robust authentication mechanisms can effectively safeguard against MiTM attacks. In addition, Intrusion Detection Systems (IDS) and Intrusion Prevention Systems (IPS) can be employed to monitor network traffic for any signs of MiTM attacks. Afterwards, proactive measures can be taken to prevent these attacks. Moreover, dynamic context-aware IDS-IPS can be deployed to detect and prevent unexpected changes in the behavior of the system. This includes those behaviors indicative of MiTM cyber-attacks [56]. Similarly, encryption is instrumental in the securing of the data layer of an Advanced Metering Infrastructure (AMI) system, preserving data privacy and protecting it from attacks such as MiTM [1].

• *Smart meter physical capture*

**Fig. 6:**
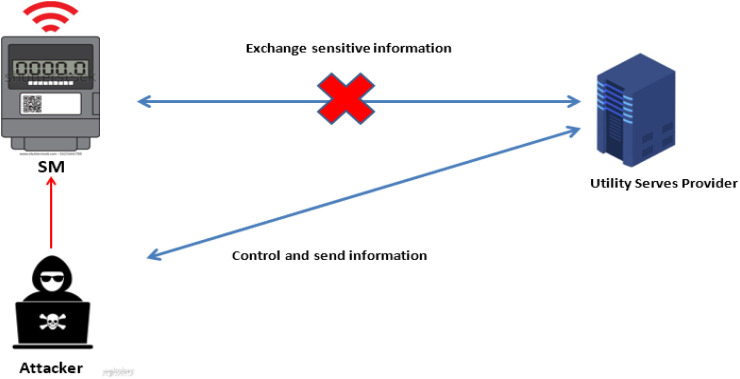
Smart meter physical capture cyber-attack.

**Mechanism:** in this attack, an adversary tries to obtain sensitive data or tamper with the device by physically gaining access to the smart meter. This attack can be executed in a number of ways, including cracking open the meter’s casing, getting over security measures with specialized tools, or taking advantage of security holes in the meter's hardware or software. Fig. 6 gives an illustration of smart meter physical capture cyber-attack. After gaining physical access to the smart meter, an adversary can attempt to extract private data, user information or encryption keys. The attacker might also try to alter the firmware or software of the smart meter to change the transmitted data. In a nutshell, this attack can facilitate data theft, illegal access to the smart grid network or even physical harm to the meter or the grid infrastructure [41].

**The effect on the smart grid:** As already explained, this attack enables the adversary to have physical access to the smart meter. Afterwards, attempts may be made to steal smart meter data, modify its firmware, or disrupt its operation. These attacks can have a significant impact on the smart meter itself, as well as the wider smart grid infrastructure [41].

**Solution and limitation:** One of the most effective techniques for mitigating this risk is to implement tamper-resistant hardware and securing smart meter enclosures. This renders it extremely difficult for unauthorized users to gain access to the internal smart meter components. Additionally, the use of strong physical security measures such as locks and seals can help prevent tampering or unauthorized access to the smart meters [41]. Since the smart meter has limited resources, it can only support lightweight encryption methods. As such, smart meters are exposed to data security preaches upon their physical capture. This is because data extraction can take place by compromising the weak encryption methods. Although the smart meter can be physically taken down and its parameters extracted, these values cannot be utilized to assume the identity of any non-compromised smart meter or that of Neighborhood Area Network (NAN) [58] .

• *Denial- of-Service (DoS)*

**Fig. 7:**
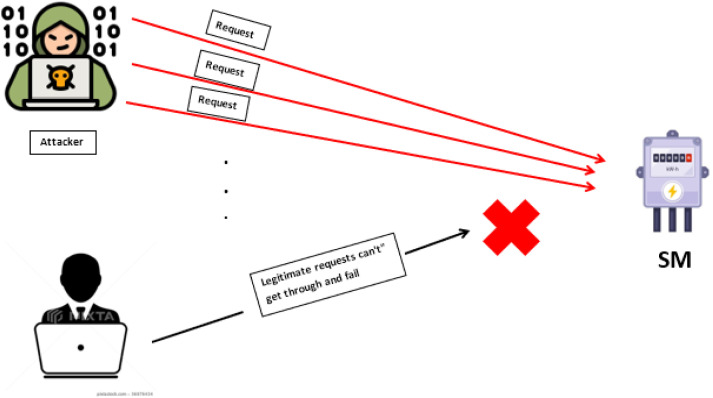
DoS attack in smart grid network.

**Mechanism:** These attacks usually overwhelm a target with malicious requests so much that legitimate requests cannot be served. Effectively, legitimate users are denied access to their entitled resources or services [2]. As shown in Fig. 7, DoS attacks disrupt IoT networks by flooding them with illegitimate packets and directing all available network resources towards serving these packets. Basically, DoS attacks are utilized to interfere with the process of transferring data between nodes. This is attained through jamming radio signals or introducing a fraudulent and malicious node into the network. In particular, DoS attacks targeting the communication between control centers and smart meter equipment may prevent signals from getting to their intended targets in time. This can hinder the control center’s ability to monitor the state of the grid and ultimately cause instability. On the other hand, a DDoS attack can be instigated by the attackers using a large number of devices (including botnets). To conceal their identity, the adversaries deploy spoofed IP addresses. The most pertinent DoS active attack methods against the SG that could result in grid instability or unavailability are compiled [59].

**The effect on the smart grid:** A successful DoS attack can have a severe impact on intelligence counters such as disabling them, reduce their performance, or corrupt their data. This renders it impossible for operators to have a glance at the entire power system. Consequently, improper decision-making by the operators crops in [60]. DoS attacks have a simple design, user interface and do not require much knowledge, skill, or resources to operate [10]. As such, they are easy to launch and cause havoc on the targeted system. Even though they do not result in any loss of sensitive data, these attacks can seriously harm the system in terms of operating expenses. As such, DoS attacks pose the greatest risk to IoT-based smart grid systems. This is worsened by the fact that they can be carried out at several layers of the smart grid. For instance, at the application layer, DoS attacks can deplete the system’s memory, computational capacity, or bandwidth. This is achieved through bombarding the system with too frequent requests. Since smart grid communication devices have limited processing power, application layer DoS attacks could potentially target them and render them unresponsive [56,61].

**Solution and limitations:** The authors in [31] propose strong and lightweight key agreement and authentication LSPA protocol built on Elliptic Curve Cryptography (ECC). For secure communication, the LSPA protocol uses elliptic curve encryption. Compared to other encryption algorithms, ECC provides strong security at shorter key lengths. This posts it as a suitable encryption technique for resource-limited environments such as the smart grid. The protocol in [31] comprises of multiple steps that carry out authentication and session key agreement between network entities [31]. It is lightweight, robust, and incorporates timestamps that are frequently checked at the receiver terminal. The session is basically terminated if these freshness checks flop. As explained in [59], DoS attacks do not alter the smart grid infrastructure or change its data.

• *Distributed Denial of Service (DDoS)*

**Fig. 8:**
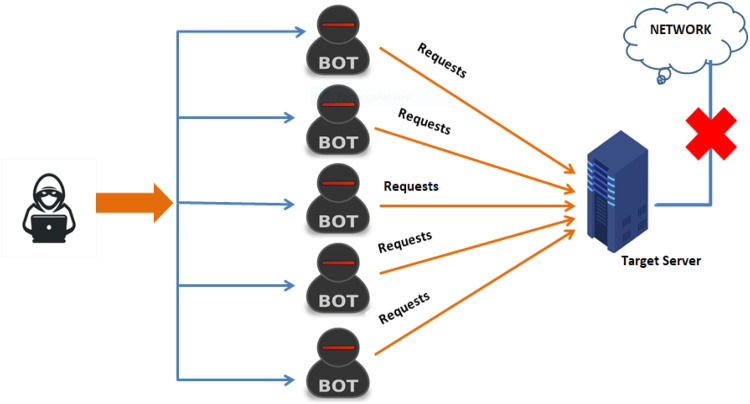
DDoS in smart grid.

**Mechanism:** This is a type of DoS attack that is more effective and disastrous since it deploys numerous botnets or robotic networks to launch the attacks. Utilizing thousands of robots (zombies), it transmits hundreds of requests per second to the target system. This results in the inundation of the system with numerous bogus requests, effectively blocking requests from legitimate users [57,62]. Typically, DDoS attack designers coordinate a large pool of compromised devices (mostly IoTs devices), to send an excessive amount of traffic to the target system [63]. Fig. 8 gives an illustration of a classical DDoS attack in the smart grid environment.

**The effect on the smart grid:** an effectual DDoS attack can affect the data centers and servers that store and process massive amounts of data generated by smart grid components. This can potentially result in both data loss and service disruptions. They can also interfere with the communication between smart meters and the utility company’s systems, causing inaccurate meter readings, billing problems, and potential disruptions for consumers [62,64].

**Solution and limitations:** There are multiple methods for identifying these attacks: conventional techniques, signature-based disclosure, and odds-based identification. Conventional techniques are centered on traffic volume measurement and hence a DDoS attack is inferred when the measured traffic volume rises above a predetermined threshold. On the hand, signature-based disclosure techniques identify DDoS threats by utilizing attack signatures stored in their databases. This process entails monitoring traffic patterns and contrasting them against previously collected signatures. However, this method can only identify attacks whose signatures are already stored in its database. In addition, it has low detection accuracy and high frequencies of false alarms.

In odds-based identification strategy, a basic profile is created by gathering normal traffic behavior over a predefined period of time. Here, an anomaly is regarded as any incoming pattern that deviates from the baseline range. The disadvantage of this method is that it has relatively low detection speed and high overheads [65] . As explained in [62], Web Application Firewall (WAF) is a viable security measure that can be utilized to safeguard the smart grid from DDoS attacks. The authors in [66] explain that it is generally rare for a DDoS attack on smart grids to cause data breach, destruction of physical hardware, or data manipulation.

• *Replay attacks Mechanism*

**Fig. 9:**
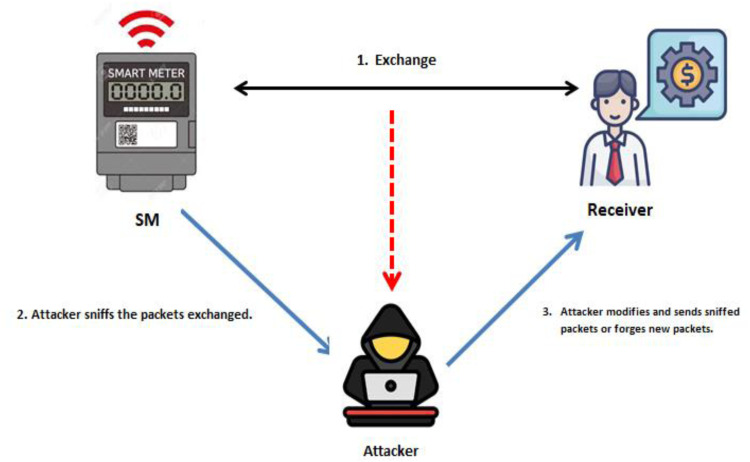
Replay attack in smart grid.

This is an active attack in which adversaries intercept communication between two entities such as smart meter and service provider and attempts to retransmit the intercepted messages later on. This attack enables the adversary to record, observe, copy, and re-transmit a portion of the copied data [62]. In a nutshell, a replay attack represents a sophisticated exploitation technique employed by malicious actors, whose objective is gaining unauthorized access to a system through the retransmission of previously intercepted or transmitted data. This form of cyber threat highlights the need for resilient security measures and rigorous data integrity mechanisms across information systems and networks. As discussed in [7] replays pose a serious risk to the security of both data and network infrastructure. A typical replay attack in smart grid is shown in Fig. 9.

**The effect on the smart grid:** In the context of smart meters, replay attacks can lead to the retransmission of metering data potentially resulting in inaccurate billing and consumption records. This can have devastating effects on the financial aspects of energy distribution and consumer billing. In addition, these attacks can result in the retransmission of sensitive information and disruption of the flow of critical data between grid components [62]. All these issues can negatively impact the communication networks used within the smart energy networks.

**Solution and limitations:** The decryption of a message increases its counter number, allowing for the detection of lost messages. In addition, it can prevent replay attacks through the detection of re-transmission attempts [67]. Moreover, authors in [57] explain that Authentication Header (AH) protocol can protect against replay attacks. Basically, the sender appends the current timestamp *t* to message *m* prior to its transmission to the receiver end. When the receiver obtains message *m*, it verifies the validity of timestamp *t* against its current timestamp t* and maximum permissible transmission latency Δ*t*. The main drawback of this method is its requirement for synchronized clocks of the sender and receiver [68].

• *Forgery*

**Fig. 10:**
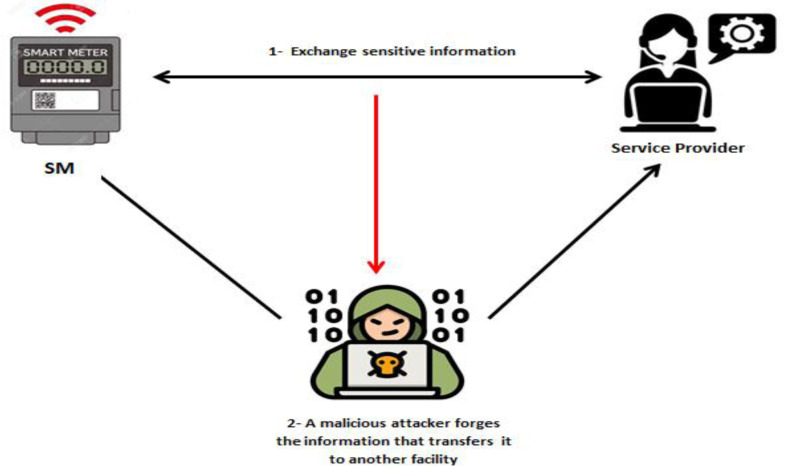
Forgery in smart grids.

**Mechanism:** This attack attempts to falsify a message’s digital signature devoid of access to the signer’s private key. To compromise the network, the malicious attribute server counterfeits keys and legitimate user signatures and distributes them to other parties [69] . A typical forgery attack is depicted in Fig. 10.

**The effects on the smart grid:** Power generation, transmission, and distribution can all be disrupted by forged data, which can also be used to compromise the smart grid functionality. This can result in isolated or even extensive outages which can negatively affect residences, commercial buildings, and the provision of necessary services. In addition, messages can be counterfeited by adversaries to activate false alerts or tamper with smart meter measurements. This can result in unstable grid circumstances resulting in monetary losses for both consumers and energy providers [70].

**Solution and limitation:** One of the most effective ways of combating forgery attacks in smart grid systems is deploying robust authentication techniques such as digital signatures and message authentication codes (MACs). These methods guarantee the integrity and validity of the transferred data in these smart energy networks. For instance, digital signatures permit the data to be signed by the sender and verified by the recipient, preventing data manipulation during transmission. However, these solutions incur high computational overheads associated with the generation and verification of digital signatures and MACs. This can negatively affect the performance of smart grid systems [54].

• *Impersonation attacks*

**Fig. 11:**
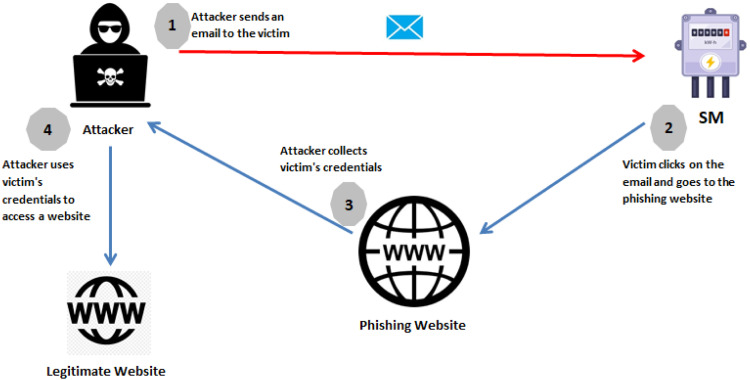
Impersonation attack in smart grids.

**Mechanism:** An impersonation attack is a specialized form of phishing threat in which an adversary masquerades as another individual or entity with the aim of obtaining confidential information from unsuspecting parties. In the smart grid scenarios the attacker can spoof someone’s identity and deceive him/her into paying for energy usage [25]. This type of attack represents a significant security violation, highlighting the need for strong safeguards and authentication protocols in information systems and network [11,71]. Fig. 11 gives an illustration of a typical impersonation attack in the smart grid environment.

**The effect on the smart grid:** Through this attack, adversaries can masquerade as legitimate users, spoofing their identities and demanding payment for energy consumption [25].

**Solution and limitations:** In [72], the authors develop a Low-Latency Authentication and Key Exchange Protocol (LLAKEP) based on the one way hash function and ECC point multiplication. Apart from this technique, blockchain-enabled privacy-preserving scheme (BPPS) is a secure, key-agreed-upon system that can uphold integrity of demand-response data. This protocol is shown to mitigate various attacks that are common in the smart energy networks. Although ECC is less costly compared with asymmetric approaches such as Rivest-Shamir-Adleman (RSA), scheme based on ECC are unsuitable for small lightweight applications typical in smart grids due to operations such as scalar multiplications [73,74].

• *Eavesdropping Mechanism*

**Fig. 12:**
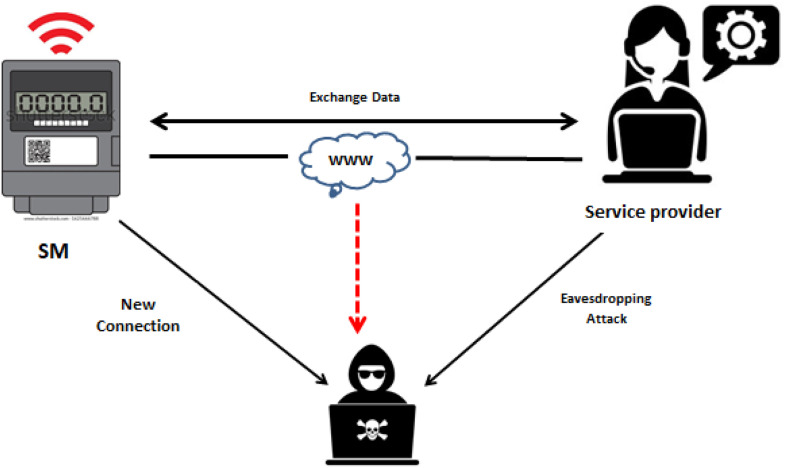
Eavesdropping attack in the smart grid.

This is a passive attack which takes place when an adversary intercepts the communications between nodes over a communication channel [56]. It enables malicious entities to secretly monitor wireless connections and their exchanged data payloads. The objective of the adversaries in this attack is to intercept network traffic and decipher the content of messages transmitted across the network. This form of attack represents a serious security concern in the realm of wireless communication, warranting heightened attention and robust protective measures [75]. Particularly, adversaries can deploy this attack to listen to or record data exchanged between smart grid components such as between smart meter and client apps. Eavesdropping compromises system privacy and can facilitate illicit activities such as identity theft and smart meter data theft. Using the stolen data, adversaries can study customer behaviours and system characteristics [52]. In Fig. 12a sample eavesdropping attack on the smart grid network is illustrated.

**The effect on the smart grid:** Eavesdropping on data transmission between the smart meter and customer applications compromises system privacy. In addition, it allows the execution of illegitimate actions such as data theft and identity fraud [76]. By listening to conversations, attackers can gain access to confidential information [77]. In addition to violating network privacy, eavesdropping can enable adversaries to access other valuable data such as billing information and power consumption reports. On the other hand, phishing attacks pose a serious risk since consumers might not discard their bills or receipts in a proper manner. This gives adversaries access to data they might use to manipulate the communication, forge messages or gather vital information [25].

**Solution and limitations:** the Virtual Private Networks (VPNs) can be deployed to protect communication and identity by supporting confidentiality and encryption of data. This can effectively be utilized to prevent eavesdropping of the messages when traversing the internet [57]. Encrypting SCADA communications using a secure key is another viable technique that can safeguard against eavesdropping [78]. Moreover, strong authentication mechanisms such as two-factor authentication can make it difficult for attackers to gain access to the data even if a tapping attack is successful. Therefore, provided that strong encryption techniques are deployed to protect communications and data within the smart network, eavesdropping attacks cannot facilitate the discerning or access to protected information [52].

• *Spoofing attacks*

**Fig. 13:**
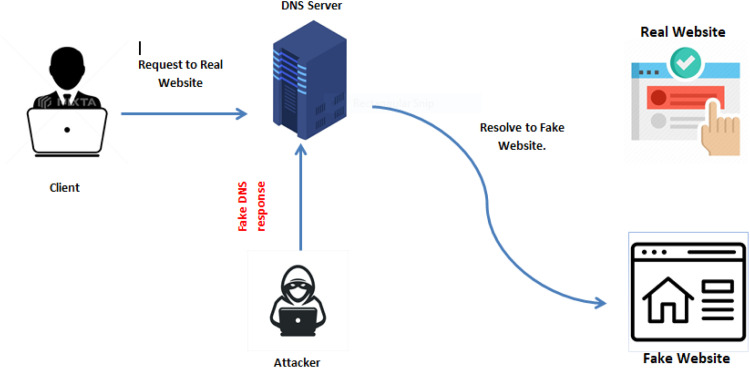
Spoofing attack in smart grids.

**Mechanism:** In smart grids, the primary objective of this attack is to interfere with the network traffic that the dispersed smart meters are measuring. The implications of this attack are varied, and include the extension or shortening of the source route, the introduction of bogus data into the transferred messages, and the maintenance of existing routing loops. Basically, spoofers pose as legitimate nodes with the aim of deceiving other nodes in the network. A successful spoofing attack can lead to the compromise of smart grid security, stability, and functionality. Fig. 13 gives a depiction of a smart grid spoofing attack.

**The impact on the smart grid:** Spoofing can have a significant negative effect on the components of the smart grid system. Through spoofing, adversaries can obtain secret values that they need to impersonate legitimate entities or devices within the smart grid. Afterwards, attackers can gain unauthorized access to sensitive information, manipulate data and disrupt the normal operation of the smart grid [76].

**Solution and limitations**: In [76], the authors implement a mutual authentication scheme among smart grid entities and components to prevent spoofing attacks. The developed protocol ensures that adversaries cannot acquire encryption or decryption keys or interfere with authentication processes. To effectively protect the infrastructure from spoofing attacks, this scheme can be reinforced by incorporating advanced security mechanisms such as communication encryption and digital signatures [39].

• *Insider attacks*

**Fig. 14:**
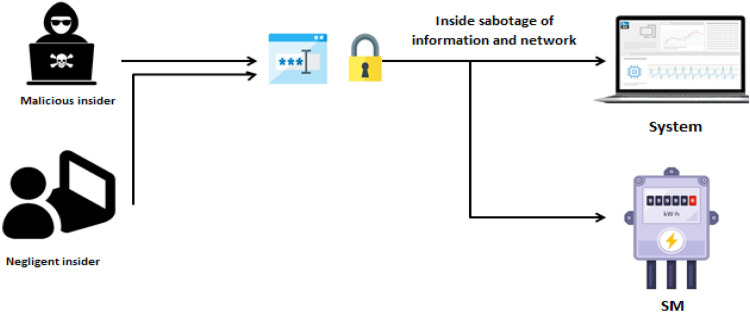
Insider threat in the smart grid network.

**Mechanism:** This security threat occurs when a person with legitimate access to organizational systems, data, or network intentionally undermines the confidentiality, integrity, or availability of sensitive information or resources for personal benefit or malicious purposes. Since they originate from within the trusted circle of an organization such attacks pose a unique and significant security challenge. This calls for enhanced monitoring and advanced security measures to detect and prevent them. Such measures may include regular personnel training and robust monitoring mechanisms to mitigate their impact [14]. Fig. 14 presents a typical insider threat in the smart grid ecosystem.

**The effect on the smart grid:** Insider threats on the data produced by different smart grid devices can potentially lead to the improper handling of different forecasting models, whose goal is to balance demand response with user satisfaction. For effectual management of demand response and data analytics, a variety of known vulnerabilities that can facilitate insider attacks must be eliminated. This can go a long way in ensuring that insiders with permission to access critical parts of the smart grid systems cannot misuse their power to compromise grid security. In addition, user privacy can be compromised if these insiders retrieve sensitive and vital information that should not be accessed by unauthorized parties [79].

**Solution and limitations:** The authors in [77] explain that regular data backup and effective security policies are essential for the protection of smart grids against insider attacks, as well as the restoration of grid operations upon compromise. To ensure enhanced protection, data backups should be stored securely and at a distance from the main business location [77] . In [80], the author suggests the use of electroencephalogram (EEG) for the detection and resolving of human emotions. Basically, EEG measure the signals generated by electron movement across the brain neural system, including the Delta band. The emotive insights obtained from EEG device are then utilized to identify abnormal EEG signals, which are indicative of insider threats. Thereafter, these insiders can be evaluated in terms of fitness for duty. To eliminate noise from physical movements and blinking, this detection system deploys deep learning and machine learning algorithms. The evaluation results show that this approach is cost effective. To help detect unauthorized activities within the network, rigorous mechanisms for managing access and controlling powers within the smart grid network must be implemented. In addition, advanced security monitoring and detection systems need to be deployed to restrict the ability of an internal attacker to access sensitive information or carry out malicious activities [81].

• *Ephemeral Secret Leakage (ESL)*

**Fig. 15:**
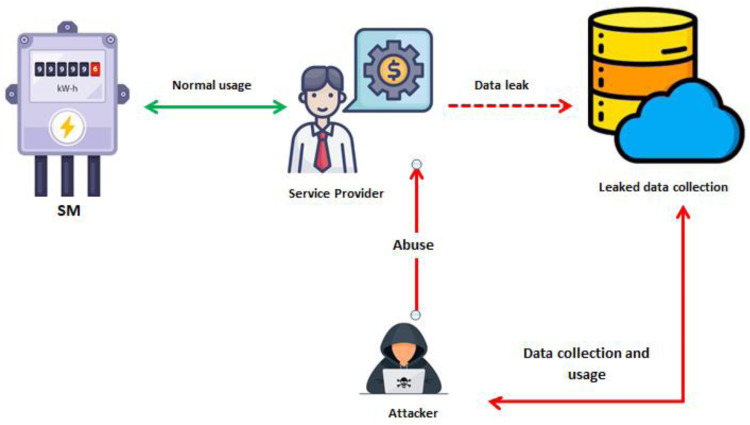
ESL cyber-attack in smart grids.

**Mechanism:** This is a cyber-attack that targets smart grids where an attacker exploits vulnerabilities in smart grid devices to steal ephemeral secrets such as cryptographic keys or session tokens. Afterwards, these secrets are utilized to gain unauthorized access to the smart grid network and disrupt its operations. Since they can be difficult to detect and prevent, ESL attacks are particularly dangerous for smart grids. In networked systems, ephemeral secrets are typically used for a short period of time, after which they are destroyed. This makes it difficult to log their use and identify suspicious activity [82]. A typical ephemeral secret leakage attack is demonstrated in Fig. 15.

**The impact on the smart grid:** The SGs rely on the exchange of sensitive information between their network control devices. This sensitive information includes the encryption keys used for securing the communications among the devices. Through the ESL attacks, adversaries are able to leak these encryption keys. Thereafter, these encryption keys are utilized to decrypt communications among the network controllers. This can lead to network disruption or even power outages.

**Solution and limitations**: The defense mechanisms against ESL attacks revolve around the use of cryptographic techniques to protect the ephemeral secrets and prevent unauthorized access to sensitive information. In addition, a session key (SK) that depends on ephemeral secrets and long-term secrets can be implemented. This ensures that even if the short-term or long term secrets are compromised, deriving the session key remains computationally difficult for an attacker. On the flip-side, potential limitations include high computational complexity of the cryptographic operations involved, the reliance on the secrecy of long term secrets, and the possibility of new cryptographic vulnerabilities emerging over time [82].

• *Privileged insider attacks*

**Fig. 16:**
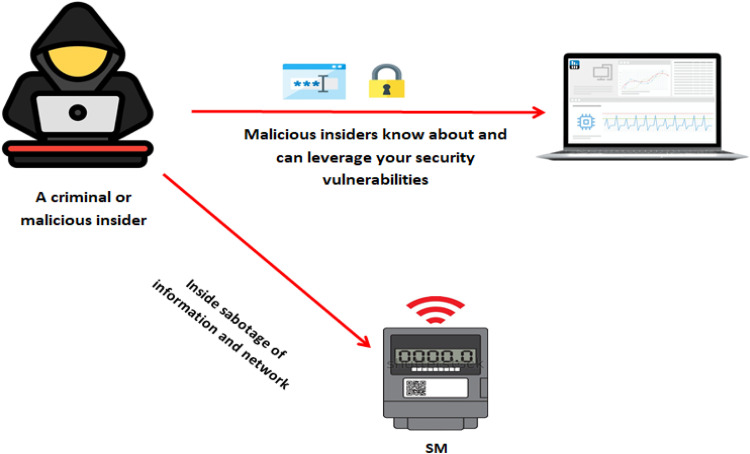
Privileged insider attack in smart grid.

**Mechanism:** This attack is performed by some privileged user, who has access to the registration information of various users and devices. This attack is extremely harder to defend and has more adverse impacts since the adversaries have access to sensitive information [83]. A typical privileged insider attack is demonstrated in Fig. 16.

**The effect on the smart grid:** malicious activities by privileged insider attackers can result in **blackouts, power outages or service** disruptions. In addition, adversaries can perform **data manipulations,** tamper with or alter crucial data such as energy consumption information or network settings. This can result into compromised decision making process that can potentially cause operational instability or blackouts [56] .

**Solution and limitations**: In [72], the authors introduce the LLAKEP+ protocol that utilized a group of cryptographic building blocks such as elliptic curve cryptography-based point multiplication, and one way hashing function. The authors carry out thorough security analysis which demonstrates that the proposed scheme defends numerous smart grid threats.

• *Guessing attacks*

**Fig. 17:**
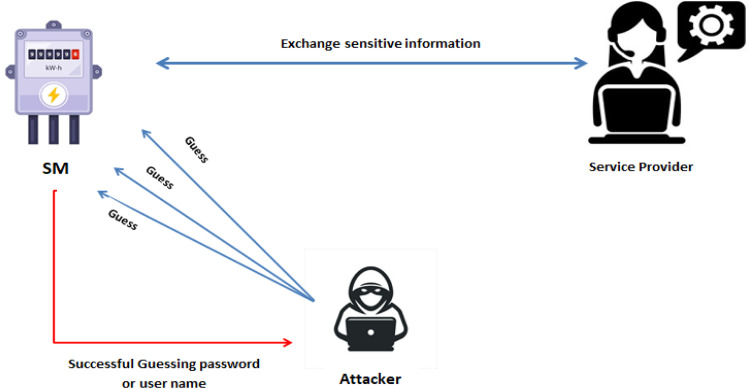
Guessing attack in smart grids.

**Mechanism:** In this security threat, an adversary carries out multiple login attempts by systematically guessing potential values of login credentials. The ultimate goal is to gain unauthorized access to the target system. Basically, this attack is regarded as being successful when the attacker manages to guess the correct password. Here, the attacker is convinced that several password guessing attempts can finally yield the correct value. As such, the use of low entropy and weak passwords increases the success probability of this attack [84]. Fig. 17 demonstrates a typical guessing attack.

**The impact on the smart grid**: By attempting to guess usernames, passwords or other authentication credentials, attackers can potentially gain unauthorized access to smart grid systems. Any successful guessing attack can results in tampering with energy readings, meter data or other crucial information. This can effectively disrupt grid operations, causing power disruptions or blackouts [12,85].

**Solution and limitation:** The utilization of lightweight cryptographic primitives such as bitwise Exclusive OR (XOR), one‑way hash functions, and concatenation is among the most viable solutions for guessing attacks [84]. The authors in [86] introduce a technique to thwart guessing attacks by making the client select a complicated password or by lengthening the size of the generated key from the password. This method can effectively minimize the impact of such an attack. However, it is not possible to stop every password guessing or social engineering attacks, not even with the developed countermeasure. Nevertheless, guessing attacks can sometimes be ineffective in accessing smart grids systems especially when effective security practices are deployed, such as using strong passwords, updating software regularly, and implementing advanced protection technologies.

• *De-synchronization attacks*

**Fig. 18:**
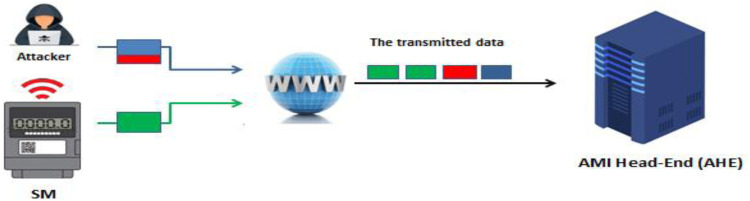
De-synchronization attack in smart grids.

**Mechanism:** The network participants that must update their values concurrently may be at risk of a desynchronization attack. In this attack, the adversary modifies the exchanged data such that it is impossible for the second party to update its values at the same time that the first party updates its desired values [87]. In the smart grid environment, the adversary can permanently break key timing by partially blocking the communications between Advanced Metering Infrastructure (AMI) Head-End (AHE) and the counter. As such, these network entities are unable to communicate with one another. Typically, when one party modifies its key, it must update the other party about these changes. However, if the attacker discards these update messages, the other party would not be aware of these changes. This will completely block any communication between these network participants [88]. Fig. 18 gives an illustration of a typical de-synchronization attack in smart grids.

**The effects on the smart grid:** Security protocols that require synchronization between two communication entities are seriously threatened by de-synchronization attacks. There are two mechanisms deployed to initiate the de-synchronization attacks. The first involves masquerading as the power management side. Thereafter, the attacker constructs arbitrary messages that are then sent to the smart meter. This effectively increases the counters on the smart meter by one while keeping the counter at the power management side unchanged. Subsequently, the power management side and the smart meter are unable to establish any secure contact. This serves to disrupt demand response and other intelligent applications. The other form of de-synchronization attack involves sending messages to smart meter from the management end. In this instance, the counter on the power management side is incremented by one, while the smart meter counter remains unchanged. As such, the smart meter and the management sides cannot agree on the same group session key, barring further communication [89]. **Solution and limitations:** To mitigate de-synchronization threats, a Scalable Key Management (SKM) scheme has been developed. This technique addresses de-synchronization attack by protecting the group key rekeying messages with MAC codes, making it immune to de-synchronization attacks [89]. As explained in [18], implementing a de-synchronization attack in the smart grid environment is complex and challenging. This is due to the distributed nature of the grid components, as well the heterogeneity of the various communication protocols and devices involved [88] .

• *Counter Synchronization Vulnerabilities in AMI and Smart Grid Protocols*

The principal authentication mechanisms for Advanced Metering Infrastructure (AMI) and Smart Grid utilize synchronised counters, sequence numbers, timestamps, and nonce-based state variables for replay prevention and session key management. These mechanisms are often used for AMI communication architectures and light-weight IoT-orientated smart grid protocols based on standards and technologies such as ZigBee Smart Energy Profile (SEP), DLMS/COSEM, IEC 62,351-compliant secure communications, and proprietary AMI key management systems. Usually, these systems have synchronised counters that are incremented after successful authentication exchanges or key updates to ensure they are fresh and to avoid replay threats. An attacker does not require complete compromise of the authentication mechanism to manipulate synchronization states; rather, desynchronization can realistically result from selective packet dropping, replay suppression, message delay, channel jamming, or disruption of rekeying messages in unreliable wireless communication contexts. During the update process, the attacker can block or modify the packets related to synchronization so that one entity (e.g., the smart meter) advances its counter while the other entity (e.g., the AMI headend or utility server) remains in an outdated state; therefore, no successful session key agreement can be reached.

The proposed Scalable Key Management (SKM) approach enhances scalability for large-scale AMI deployments by using hierarchical or group-based key management rather than maintaining independent paired keys for each smart meter. This reduces the overheads on storage, computation and communication, because messages for group rekeying can be efficiently distributed to multiple devices simultaneously using lightweight cryptographic operations such as hash functions and message authentication codes (MACs). Existing AMI and smart grid standards address recovery from desynchronization attacks primarily through resynchronization protocols that rely on retransmission requests, counter reset mechanisms, challenge-response interactions, timestamp verification, or periodic re-authentication. Furthermore, centralized AMI head-end systems may periodically initiate secure resynchronization sessions or disseminate new session keys upon detecting synchronization faults. Consequently, while desynchronization attacks can impede secure communication and session initiation, contemporary AMI systems generally have fallback synchronization and recovery protocols to re-establish secure communication continuity while reducing service disruption.

## Phase 3: cybersecurity threat and attack analysis

During this phase, we investigated key cyber threats targeting smart grids and IoT-enabled infrastructures. The analysed attacks were: man-in-the-middle, replay, denial-of-service (DoS), ransomware propagation, malware infiltration, false data injection, desynchronization attack and insider intrusion into SCADA. The study evaluated each attack category by its methodology, the conditions under which it can be exploited (how), its operational impact, communication vulnerabilities and its potential impacts on grid stability, data integrity, privacy and continuity of service.

• *Structured Query Language Injection Attack (SQLIA)*

**Fig. 19:**
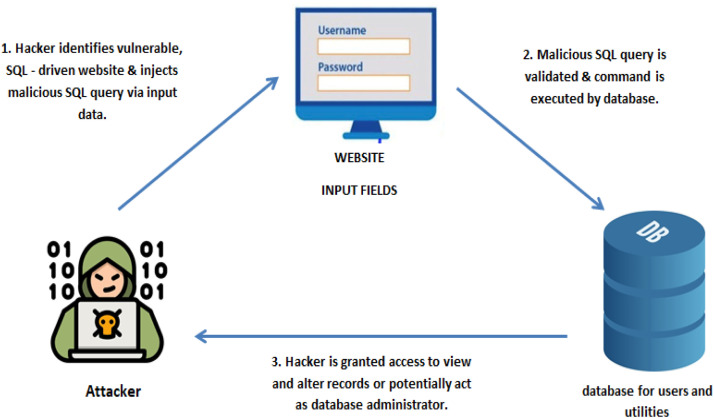
SQLI attack in the smart grid.

**Mechanism:** These attacks are the biggest risks to web application security. A typical SQLIA involves tricking the server in a manner that allows the malicious code to access the database. Basically, SQLIA inserts malicious code that alters database information which can cause serious compromise to online applications. By deploying user input to insert malicious code into the regular commands, the adversary can exploit database information to control or manage online applications. It involves utilizing SQL to issue a database query that tricks the server into executing an injected query that reveals the database information [90]. For instance, using a website search box on the client-side interface of the program, the attacker can enter the query that targets particular databases [62]. An illustration of a conventional SQLIA is depicted in Fig. 19.

**The effect on the smart grid:** Using script commands, SQL injection attacks can change databases or insert malicious content. By inserting malicious queries into the database, SQL injection attacks can enable the adversaries to take over the system, add or alter existing data, remove data, or gain control of this data. This might interfere with smart grid operations and ultimately cause blackouts. Typically, electricity consumption data is continuously sent by the smart meters to the utility service provider, where they are stored in databases. If user queries are not properly validated before they are executed, SQL injections can take place [56].

**Solution and limitations:** To mitigate against SQLIAs, methods which rely on encoding all the data in databases have been developed. This involves adding a special character to prevent the process from going backward. Afterwards, a permutation encoding method is deployed. Here, the permutation method encompasses the modification of bit location inside three characters prior to using radix encoding. Basically, permutation is based on the primitive root value and is concealed through encoding. To implement and test the suggested approach, PHP and MySQL databases are utilized. Thereafter, the obtained results are compared to those of other proposed methods. The security analysis shows that the suggested solution prevents SQLIA and safeguards database information. Since encoding saves the server from heavy calculations, it is suggested as a preventative measure against various forms of SQLIA [90]. In [91], the authors develop a defense strategy against SQL injection attacks based on searchable encryption and cryptography. In this method, every piece of user data is encrypted with a unique key. As such, all database information is enciphered using a cryptographic methodology. In order to protect privacy, the rest of the database data is encrypted using secret keys, while other database functions employ searchable encryption. To verify the user’s identity, the login procedures verify the user’s login with encrypted username from the database. To restrict access to databases and sensitive information, smart grid systems typically implement strict access controls and authentication mechanisms. This makes it harder for attackers to exploit SQL injection vulnerabilities.

• *Brute Force attacks*

**Fig. 20:**
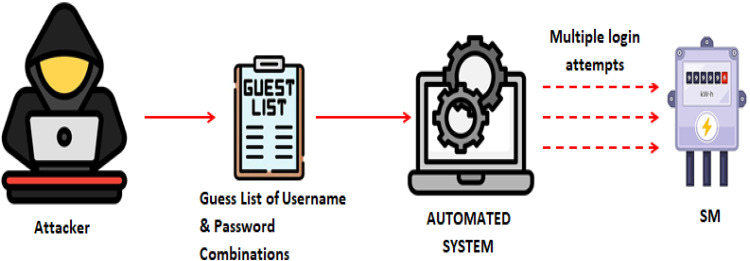
Brute Force attack in smart grids.

**Mechanism:** Although brute force attacks assume different forms, they usually involve the attacker setting up predefined values, sending requests to a server with those values, and then examining the response. In order to maximize efficiency, an adversary may employ a dictionary attack (with or without mutations) or a conventional brute-force attack with certain character classes, such as alphanumeric, special, and case (sensitive). For a particular method, the adversary can deploy the number of tries, system efficiency, and the predicted attack system efficiency to determine the approximate time required to submit all selected predefined values [92]. As explained in [12], the main targets of the brute force attack are security and privacy of the collected smart meter data. An illustration of a typical brute force attack in smart grids is given in Fig. 20.

**The effects on the smart grid:** Brute force attacks can have significant impacts and consequences on the smart grid systems. For instance, they can enable the adversaries to access sensitive information such as energy consumption patterns, critical infrastructure details and personal customer data. Thereafter, this information can be used for targeted attacks or extortion. In addition, attackers can disrupt normal grid operations by overloading the system with numerous log in or data manipulation attempts resulting in power outages or full network failure [54].

**Solution and limitations**: The authors in [54] and [76] have suggested strong passwords as an effective strategy against these security threats. These systems deploy complex and lengthy passwords to increase the time required to get correct marches. Another effective technique for mitigating brute force attacks on smart grid authentication involves limiting the number of login attempts. Furthermore, multi-factor authentication can be deployed, where that biometrics and passwords are integrated to strengthen protection against brute force attacks. However, all these techniques have limitations in that they may increase the complexity and cost of the authentication process [12]. In order to make it more difficult for attackers to guess the proper key, it is crucial to utilize brute force-resistant encryption methods as well as longer encryption keys [12].

• *Repudiation threats*

**Fig. 21:**
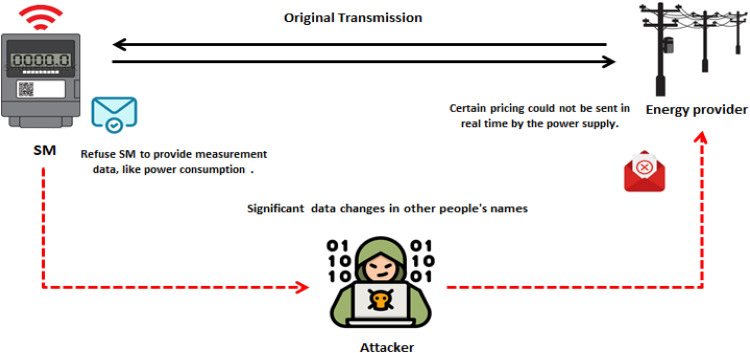
Repudiation attack in the smart grid.

**Mechanism:** These attacks are common in applications or system architectures that neglect the security of user activity tracking and logging. Just like spoofing emails, majority of repudiation attacks involve data alteration maliciously carried out on behalf of legitimate network entities. When adversaries successfully carry out this attack, the information kept in log files may be corrupted and hence rendered inaccurate or deceptive [39]. Non-repudiation services are necessary to prevent the energy provider or the smart meter (end customer) from refuting having given real-time price information or metering data about energy consumption [93]. Fig. 21 given a depiction of repudiation attack in the smart grid ecosystem.

**The effect on the smart grid:** Tampering with smart grid readings can lead to inaccurate billing. On the other hand, modified data can negatively affect the prediction of demand and resource allocation. These security threats can therefore affect energy availability, potentially causing power outages [76,94].

**Fig. 22:**
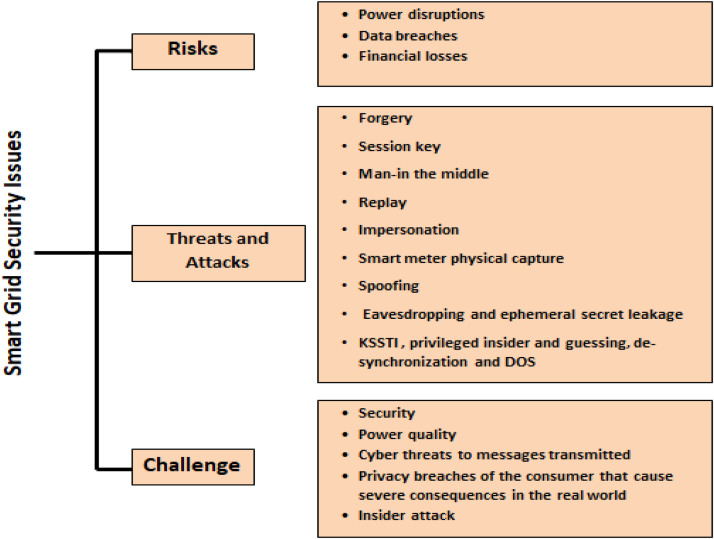
Smart grid security issues.

**Solution and limitations:** To ensure that origin data and its integrity is verifiable, digital signatures for user actions and system logs can be implemented. Basically, these security techniques uphold non repudiation. In addition, Role Based Access Control (RBAC) mechanisms can be implemented to restrict access to sensitive system logs and actions. In so doing, these security techniques ensure that only authorized entities can interact with critical data [39]. Fig. 22 gives pictorial representation of the various security issues within the smart grid.

### Attacks against scada systems

The SCADA networks are vulnerable to both internal and external attacks. Whereas internal attacks are initiated by employees or contractors, external attacks are launched by outsiders and include malware and hacking [47]. For instance, a false data injection attack can target smart meter or neighborhood area network. In addition, it can target other smart grid infrastructure, damaging measurement and monitoring subsystems, or manipulate meter and phasor measurements. Upon successful compromise, attackers can inject manipulated data into SCADA centers, bypassing data integrity checks [56].

For instance, the 2010 Stuxnet infection in Iran’s SCADA system resulted in the damage to industrial components. Stuxnet's objective was to reprogramme specific industrial control systems while concealing any modifications [14,56] . Stuxnet malware targets Siemens SCADA applications PCS 7, WinCC, and STEP7 on Microsoft Windows and Siemens S7 PLC. It spreads via USB flash drives and uses zero-day exploits to infect SIMATIC WinCC and PCS 7. It affects Programmable Logic Controller (PLC) systems with variable-frequency drives. Stuxnet disrupts the operation of the connected motors by changing their rotational speed [94]. On the other hand, a DoS attack on SCADA in Ukraine in 2015 resulted in a three-hour service interruption for customers by disconnecting substations [95]. Conventionally, SCADA networks are secured using technologies such as Virtual Private Network (VPN), Internet Protocol Security (IPSec), firewall, user and device authentication, as well as Intrusion Detection Systems (IDS) [56,96].

### Limitations of scada systems

SCADA networks play an important and essential role in various critical infrastructures. This is more pronounced in the field of electric power generation systems. The conventional SCADA networks were initially designed for closed environments, resulting in limited security attention. However, with open access networks such as the Internet becoming essential, modern SCADA networks face numerous security challenges. The resource limitations of RTUs and Intelligence Electronic Devices (IEDs) [78] curtail the deployment of complex security techniques. This necessitates the implementation of efficient key management schemes and lightweight ciphers for SCADA communications. In addition, there is need for lightweight cryptographic algorithms and protocols for secure data exchanges in these systems.

## Phase 4: comparative analysis of communication and security protocols

The fourth phase was a comparative analysis of communication protocols and security techniques in smart grids and IoT environments. We study protocols such as HTTP, MQTT, CoAP, ZigBee, 6LoWPAN and TLS/DTLS proposals for ECC-based authentication schemes in addition to lightweight key management protocols regarding computation complexity, communication overheads, scalability and interoperability features of the proposed scheme, applicability profiles (suitability for resource-limited devices including smart meters, sensors and edge nodes).

### IoTs and smart grid-based communication protocols

**Fig. 23:**
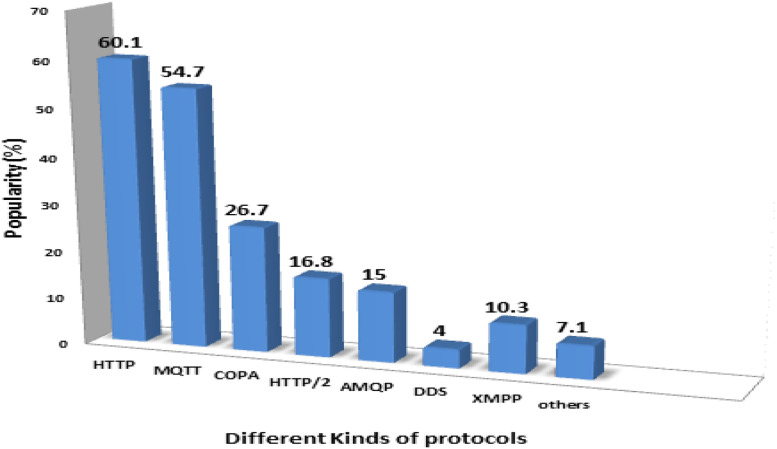
Popularity of different kinds of protocols [97].

In this section, the most common protocols and their percentage popularity are discussed. This popularity percentage is then summarized in Fig. 23.

#### Hypertext transfer protocol (HTTP)

This is the most widely used web protocol and is considered as the foundation of the internet [45] .In IoTs, HTTP is very popular, where it is deployed at the application layer to facilitate easy user access through the World Wide Web (WWW) and hypertexts. Its enhanced version (HTTP/2) was published in 2015 and supports maximum web browsers. One of HTTP’s primary advantages is that it is memory efficient, owing to the limited number of simultaneous networks [42]. On the flip side, its optimization is not possible for mobile devices [42]. In addition, attacks such as HTTP flood, Cross-Site Scripting (XSS), and SQL injections can overwhelm web servers, steal sensitive information, and modify database access. These attacks can be difficult to defend against. This is due to their reliance on multiple sources, as well as the difficulty in distinguishing legitimate traffic from bogus one [42].

#### Constrained application protocol (CoAP)

This is a lightweight protocol that was created to meet the need for a protocol appropriate for all resource-limited devices. As such, it can facilitate Machine-to-Machine (M2M) applications including automated smart homes. The devices supported by CoAP are characterized by their constrained nature in terms of Low Power and Lossy Networks) LLNs(. According to [98], this protocol is preferred due to its lightweight, low power, low resource device interoperability, simplicity for developers, and Datagram Transport Layer Security (DTLS) security capabilities [98]. In contrast to other IoTs protocols, DTLS makes CoAP lose its multicast definition, which is a prominent standard [99,100]. On the flip side, CoAP is slow in that it continuously sends an acknowledgement message until an approval message is received. In addition, it supports various asynchronous messages transmissions [42]. Despite its widespread adoption, CoAP is still susceptible to certain attacks, such as the DDoS, repudiation, and replay [45]. DDoS is particularly as it aims to consume the target system’s resources to the extent that it becomes unavailable for legitimate users [98].

#### Message queuing telemetry transport (MQTT)

This is an open standard protocol introduced in 1999 and involves the publisher, subscriber, and broker. It ensures reliable communication through brokers who cross-check authorities for topic-specific exchanges [99]. The message delivery process involves sending a message to a message broker where it is placed in a queue and then automatically delivered to all subscribed customers [101]. Basically, MQTT is a lightweight protocol for distributed sensors with three levels of QoS: level zero, level one, and level two. To maintain the connection for subscribers and receive updates, this protocol (which operates over TCP) requires the broker [99]. In a nutshell, the MQTT broker manages communication between MQTT clients and distributes messages. It can handle up to thousands connected MQTT clients simultaneously. These brokers find clients with subscriptions to the received topic [102]. Although it is easy to implement, it has limited scalability due to the involvement of the broker [42].

#### Advanced message queuing protocol (AMQP)

The protocol was introduced in 2003 and later standardized by OASIS in 2011 [99]. It consists of data exchange, queue and binding elements. Using a virtual address key, the exchange model accepts publisher messages and routes them to queues based on predefined criteria [100]. It is an asynchronous, open source protocol that enhances interoperability. The AMQP protocol prioritizes messages and supports a wide range of message structures. However, this protocol is not lightweight and hence not suitable for resource-constrained devices [99].

#### Lightweight tls/dtls optimization for resource-constrained devices

Constrained smart grids and IoT devices cope with the computational overhead of TLS or DTLS through the use of lightweight cryptographic algorithms, optimized session management, hardware acceleration and protocol simplification strategies tailored to low-power, resource-constrained environments. Original TLS implementations were built for computationally strong systems and there is significant overhead in processing certificates, handshake latency, memory usage and asymmetric cryptographic operations. To overcome such limitations in devices such as smart meters, sensors and edge nodes, modern implementations usually adopt lightweight cypher suites based on elliptic curve cryptography (ECC), pre-shared keys (PSKs), compact certificates and session resumption techniques to reduce computation and communication costs. In constrained smart grid deployments, DTLS is frequently chosen over traditional TLS because it allows for lightweight communication over UDP while offering comparable security properties. Devices bypass duplicate full handshakes with cached session states, shorter handshakes and streamlined authentication techniques to reduce overhead.

Many IoT-enabled smart meters and embedded controllers also employ cryptographic co-processors or hardware security modules to accelerate encryption, hashing and key exchange operations and to reduce energy consumption and processing latency. Protocols such as MQTT and CoAP are designed to be used over TLS/DTLS efficiently, reducing payload and network traffic sizes. Nonetheless, despite these enhancements, TLS and DTLS may still incur significant overhead on severely constrained devices, prompting numerous smart grid studies to explore lightweight authentication and key agreement protocols specifically designed for resource-limited AMI and IoT frameworks.

#### Extensible messaging and presence protocol (XMPP)

This protocol supports both synchronous and asynchronous communication models, converting Extensive Markup Language (XML) stanza messages into data. Although it is stable and customizable, it is not suitable for resource-constrained devices [42]. In addition, it is vulnerable to DoS attacks which can overwhelm servers with traffic. This renders them unavailable to legitimate users. In addition, it is defenseless against MitM attacks where adversaries intercept traffic between parties allowing them to eavesdrop or modify data. Moreover, spam attacks (which can appear legitimate) can be difficult to filter out in XMPP. On the other hand, malware attacks can install malevolent software on the user’s computer, stealing data or granting access to the attackers. Since XMPP does not encrypt all traffic [45], these security threats can result in severe consequences.

#### Common object request broker architecture (CORBA)

This system was introduced in 1991 and is a slow network protocol that supports multiple asynchronous messages or languages. It is a reliable protocol in that it continuously issues an acknowledgement message until approval is received from the other communication partner [42]. In addition, it directly addresses complex problems related to distributed computing, such as real-time or high-speed QoS, partial failures, mass communications, and causal order of events. Moreover, is very easy to implement owing to its low complexity.

#### Zero message queuing (ZeroMQ)

This is an asynchronous protocol that uses a queue for message sharing, making it suitable for high data volumes and constrained devices [42]. However, it is defenseless against attacks such as MitM, DoS, Malformed messages, replay and Sybil. Here, MitM attacks allow attackers to eavesdrop on traffic between two ZeroMQ endpoints, stealing sensitive information. On the other hand, DoS attacks flood applications, disrupting operations. On their part, malformed messages can cause endpoints to crash, while replay attacks record legitimate messages allowing attackers to authenticate or execute malicious commands. On the other hand, Sybil attacks create fake identities, spam applications or vote in misleading ways [99].

#### Data distribution service (DDS)

This is a robust protocol for Wide Area Networks (WANs) that leverages unbound infrastructure through the Internet for security and reliability. It offers real-time communication via publish-subscribe message patterns. As such, it eliminates the need for participants to know each other. It uses a domain space for communication entities, including data reader, writer, publisher and subscriber. Here, the domain participants access data based on topic and type. In spite of these positive aspects, one limitation of the DDS protocol is its complexity in terms of configuration and implementation. Another potential limitation of DDS is the high overhead associated with its real-time communication mechanisms [99]. Specifically, DDS consumes twice the bandwidth consumed by the MQTT protocol. This is because events are originated per source in real-time and not from multiple sources.

#### Open platform communications united architecture (OPC UA)

This is a combination of automation industries focusing on transport and data models. It requires firewall configurations and resource-constrained practices. Its purpose is to maintain interoperability with other operational systems through a standardized architecture. However, restrictions on broker firewall configuration lead to limited scalability [42,99].

#### Devices profile for web services (DPWS)

This was introduced in 2004 by a consortium led by Microsoft and is now an OASIS open standard**.** The DPWS consists of hosting and hosted services, providing resources-constrained implementations. In addition, it addresses security concerns in hosted services. On the flip side, the availability of multiple unconsolidated specifications (protocols, bindings, etc.) is the primary drawback of DPWS [42].

Fig. 24. Fig. 25

**Fig. 24:**
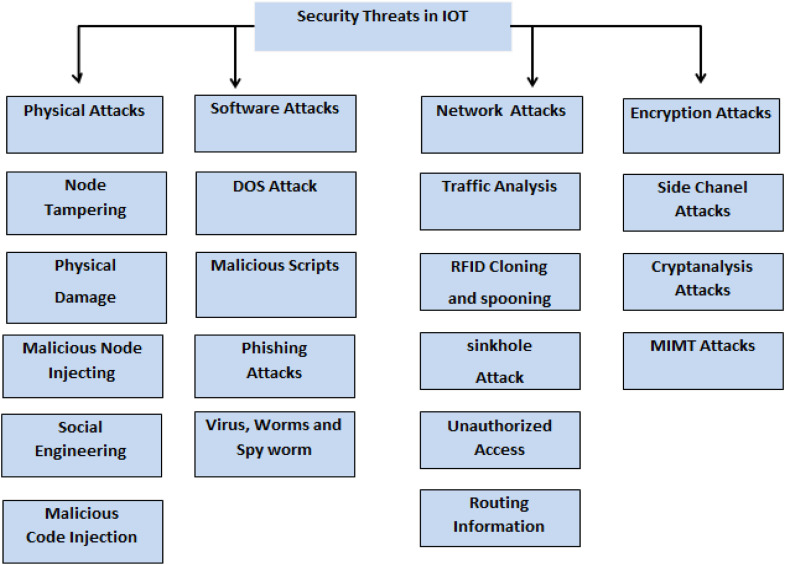
Types of attacks on IoTs integrated systems.

**Fig. 25:**
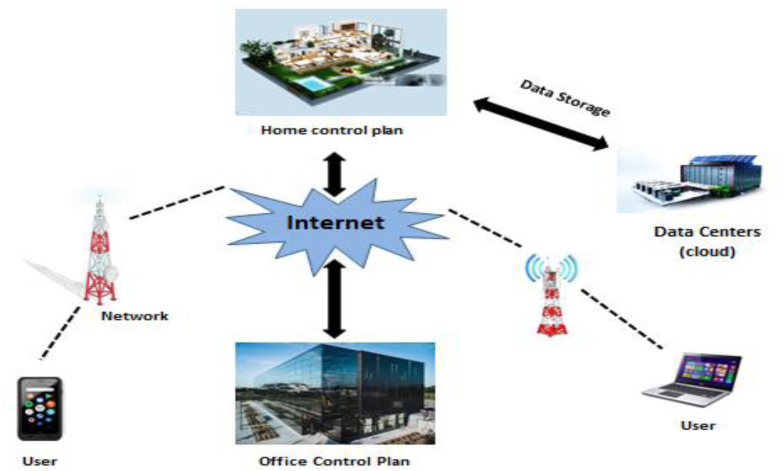
IoT-enabled advanced position-based automatic energy control.

### Communication protocol adoption in smart grid and iot deployments

This work also provides a comparison of common communication protocols for IoT and the smart grid. The indicated popularity percentages are representative of broad trends observed in the literature and industry surveys, or derived from current smart grid/IoT deployments, yet none of the figures stem from a single, standalone proprietary empirical database or a large, full-scale field measurement experiment. Percentages came from previously reported studies and from reports on adoption of protocols or comparison studies in the IoT and smart grid domain that consistently find HTTP, MQTT, CoAP, ZigBee and 6LoWPAN to be dominant communication technologies due to their interoperability properties; lightweight operation (overhead) and compatibility with constrained environments. Consequently, research here is comparative and observational rather than statistical modeling based on direct telemetry data collected from operational utility infrastructure.

MQTT has been widely used for IoT middleware and cloud-based smart grid systems due to its lightweight publish/subscribe model and asynchronous communication mechanism. In constrained wireless sensor and edge use cases, CoAP is a common protocol with respect to low-layer overhead operating over UDP. Standards such as DLMS/COSEM and IEC-related standards are widely used in utility-grade AMI infrastructures to promote metering, interoperability and grid security. While HTTP is well supported and interoperable, it is usually less appropriate for directly connecting multiple large-scale smart meters than more efficient online cloud integration layers and dashboards/administration interfaces, due to its relatively high communication and processing overhead. HTTP offers excellent interoperability and a mature security mechanism in the form of TLS. However, it has relatively high bandwidth consumption and connection establishment overhead and is latency-intensive due to its request-response nature and verbose headers. MQTT is more efficient in large-scale smart grid deployments, as its lightweight publish/subscribe model reduces communication overhead, facilitates asynchronous data delivery and allows scalable broker-based communication for real-time telemetry collection. CoAP uses compact binary headers and UDP transport to minimise overhead, making it extremely efficient for constrained edge devices and low-power wireless networks. However, the reliability and congestion management of CoAP are generally worse than TCP-based MQTT or HTTP in highly dynamic scenarios. Security-wise, HTTP is commonly used with TLS. MQTT is commonly used with TLS-secured broker communication. CoAP commonly uses DTLS for lightweight transport-layer security. However, the suitability of each protocol depends on the deployment criteria such as latency tolerance, energy constraints, bandwidth availability, scalability requirements and real-time operational reliability within the smart grid framework.

### IoT-based smart grids

The fundamental idea behind the IoTs is to rely on protocols that allow devices to connect through conventional networks. Due to the absence of real-time monitoring tools, the end user is responsible for reporting anomalous events and disruptions. Compared to conventional electric power grids, the smart grids collect more data, which necessitates increased processing. The continuous communication between the USP and the smart meter located at the user’s residence or business place accounts for this massive amount of data. The big data processing and transfer between the user and the utility service provider can be considerably aided by IoT technology. Basically, IoTs in the smart grid facilitates proactive power backup decisions, the identification of locations with excessive and insufficient power, as well as seamless and effective communication between utility control sensors and customers’ smart meter. In addition, data acquired from an IoT-based smart network can help in the optimization of error location in real time and restore it [103].

Basically, the IoT technology facilitates data collection in the smart grid network. This collection is facilitated by the enabling network infrastructure, protocols, data storage and measurement tools. The various smart grid components facilitate the generation, distribution, transmission, and consumption. In addition, IoT can be utilized in smart homes as well as for the monitoring of transmission lines and substations [42].

**Fig. 26:**
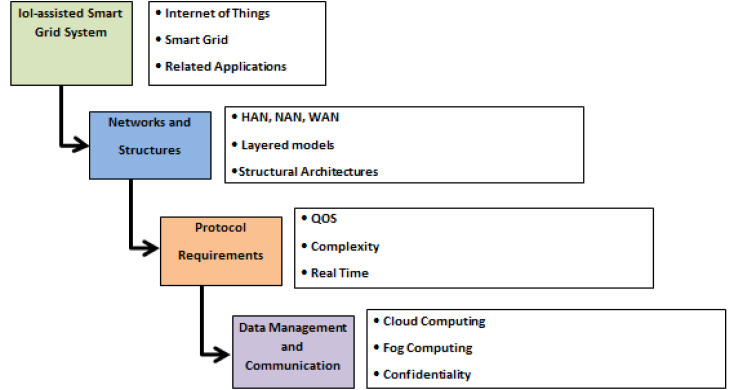
Relationship of effective elements with IoT- assisted smart grid systems.

**Fig. 27:**
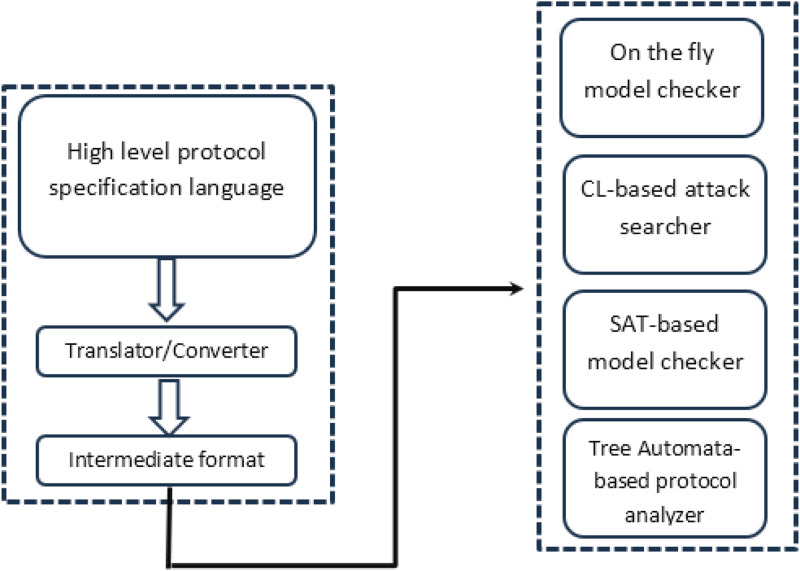
General structure of AVISPA.

Typically, the smart grid comprises of three primary networks: Home Area Network (HAN), Neighborhood Areas Network (NAN), and Wide Area Network (WAN). As the first layer, HAN is directly connected to various smart home devices, which use sensors to collect real-time data. Basically, HAN is designed to communicate smart meter information. The information processed by HAN is mostly customer-related, and is used by utilities and other smart grid components to make critical decisions [76]. On the other hand, NAN is the second layer in the smart grid that supports the flow of information between the HAN network and the third layer (Wide Area Network-WAN) network. It enables a range of applications, such as smart measurement, load management, distribution automation, pricing, power failure management, and remote control [104]. On its part, WAN facilitates real-time information gathering, real-time monitoring, control, and protection of applications. Through the support of the third layer of the smart grid, power disruptions can be averted. Compared to traditional SCADA and Power Management Systems (PMS), smart grids can support large-scale monitoring, control, and applications protection for better data resolution and faster response times [105]. As shown in Fig. 27, this permits efficient management and control of energy systems [106,107]. Since it links billions of users and gadgets in dispersed networks, IoT operates on the tenet of heterogeneity. Unfortunately, this increases its susceptibility to hacking and other security threats. Some of the well-known IoT security risks include DDoS, botnet malware, and brute force attacks [64]. The most prominent cyber-attacks on the IoT integrated system are shown in Fig. 26.

### Cloud computing

Cloud computing plays a critical role in providing the necessary infrastructure, storage, processing power, and software services to support the efficient implementation and operation of the smart grid and smart meter technologies. Particularly, the cloud platforms offer scalable and cost-effective solutions for storing and processing large amounts of data generated by smart meters and other network components. In addition, cloud computing enables real-time monitoring and analysis of energy consumption patterns. Moreover, it allows grid adaptation to changing requests and accommodates the increasing volume of data [108]. On the flip side, cloud computing requires robust security since it handles sensitive data. Specifically, cloud computing involves the transfer of sensitive data over the Internet to remote servers, which raises serious concerns about data privacy and security. In addition, cloud computing platforms send data to remote data centers for processing, which can result in undesirable elongated latencies for critical applications such as real-time monitoring and control in smart grids. These long delays can negatively affect the response of the system [109]. To ensure efficient and reliable network operations, these limitations must be addressed through various viable solutions. For instance, edge computing and fog computing can facilitate peripheral computing in smart networks. This can help address latency issues by processing data near the source. The implementation these solutions together with other security approaches can aid in effectively addressing cloud computing constraints in terms of latency, data security, scalability, and reliability. All these performance and security techniques ensure the efficient and secure operation of the smart grids [45].

The DHTG is one of the most computation intensive cloud models. As such, this cloud architecture can offer backup capabilities and basic energy usage savings. The application layer in cloud computing controls user friendly web interface systems. As shown in Fig. 27, the cloud-based architecture has an assembly control plane that resembles a tree. In addition, it has several layers of energy-saving policies at various levels, including departments, and building rooms. Here, PCs and cell phones are equipped with a variety of networking interfaces, including Bluetooth, Global Positioning System (GPS), Long Term Evolution (LTE), Wireless-Fidelity (Wi-Fi), and Third Generation (3 G) technologies [110]. These networking capabilities facilitate precise location placement in the smart grid environment. Cell phone location data is utilized to build automated control rules that can turn on and off energy-intensive appliances in residences and workplaces [1,42]. Through the smart-phones, users can dynamically modify the energy-saving rules after initial verification and authorization.

### SCADA networks

The communications and networking infrastructure of the power grids is usually represented by the SCADA subsystem. It comprises of numerous telemetry, sensing, and measurement devices integrated with a networking infrastructure. This subsystem provides the fundamental framework for data collection, communication, storage, processing, and analysis [111]. As explained in [78], SCADA networks are computer-based devices used for monitoring and controlling infrastructures and industries, integrating data acquisition, transmission, and Human Machine Interface (HMI) software for centralized processing of outputs and inputs. According to [56], the SCADA system comprises four parts, which include data interface appliances such as RTU and Programmable Logic Controller (PLC); communication network such as radio, satellite, cable and telephone; Central Master Terminal Unit (MTU); and HMI software or system.

SCADA systems are essential for large companies with automated measurement processes. They enable remote verification of real-time data using data transfer protocols such as Transmission Control Protocol/Internet Protocol (TCP/IP), allowing employees to work remotely. According to [77] and [112], SCADA systems running on Windows desktop operating systems are the primary networks used by 80% of US-based utilities and industrial systems. SCADA systems are written in low level programming languages such as C or C++ and are susceptible to buffer overflow attacks which can compromise the system [52]. Historically, SCADA systems used closed-off software. However, with the incorporation of IoTs, remote status updates have become more accessible. Nevertheless, IoTs have increased the attack surface for ransomware. This is because these networks interconnect numerous heterogeneous devices, some of which have weak security implementations. To prevent network intrusions and ransomware attacks, operating systems and software must be upgraded.

### Security challenges of legacy scada protocols in iot-integrated smart grids

This is associated with industrial SCADA communication protocols widely used in smart grid and critical infrastructure environments, including Modbus, DNP3 (Distributed Network Protocol), IEC 60,870-5–101/104, ICCP/TASE.2 and IEC 61,850-related communication frameworks. Many of the older SCADA protocols were designed for isolated operational technology (OT) environments, with a focus on availability and interoperability rather than cybersecurity. Traditional Modbus transmits commands and operational data in plain text and has no built-in authentication and early versions of DNP3 and IEC protocols offer little defence against spoofing, replay and man-in-the-middle attacks unless enhanced with secure extensions such as IEC 62,351 or Secure DNP3. These vulnerabilities allow perpetrators to wiretap or handshake between any two control centres, RTUs, PLCs, and field devices; inject packets into the communication stream or reroute traffic; and bring down communications (i.e., disrupt the communication centre). All of these are directly related to enabling the simulated attacks deep in this research including denial-of-service, spoofing, replay, ransomware propagation and unauthorized remote control. The claim that "80% of U.S. utilities rely on Windows-based SCADA systems" must be viewed in the context of prior industrial cybersecurity research and sector analyses rather than as a universally accepted industry statistic. Over the years, many utility operators have relied on Windows-based Human Machine Interfaces (HMIs), supervisory servers, engineering workstations and SCADA management systems due to their interoperability in a heterogeneous vendor ecosystem and ease of deployment. And even that number is fluid due to ongoing modernization activity and the transition from traditional architectures towards hybrid ones, as well as the increasing adoption of the cloud, Linux-based systems and embedded industrial systems. Therefore, the graph shows that Windows environments have a long history of controlling utility infrastructure, but without an empirical check against modern industry trends or government reports, it cannot be treated as a static (general) rule of thumb.

IoT restructures the classic SCADA trust perimeter, where every isolated operational environment becomes a highly interconnected cyber-physical system. In most cases, traditional SCADA architectures relied on network segmentation and air-gapped operational networks because trust points were based on outdated physical locations and limited external communication. The advancements likewise open new avenues for ransomware attacks through the introduction of impervious APIs, weak authentication, weak edge devices, misconfigured remote access services, unpatched IoT firmware/third-party SaaS integrations, zero-trust-based approaches and compromised supply chain components. Once a vulnerable IoT node or remotely connected device is compromised by attackers, lateral movement between IT and OT networks becomes easier and ransomware can spread to SCADA servers, engineering workstations and operational control systems. In turn, IoT integration deconstructs the classical boundary of trust, eliminating siloed trust assumptions in favor of always-on communication models that require enhanced authentication, segmentation, continuous monitoring and zero-trust security principles.

## Comparative analysis of advanced smart grid cyberattacks

Stuxnet and the Ukraine power grid cyberattack are both exceptionally relevant use cases for smart grid and SCADA security frameworks, yet they differ tremendously in their threat models, ranging from how the adversary gained access to the sophistication of the malware to the targeting strategy and operational goals. The adversary model considered advanced technical skill, persistent operations, a detailed understanding of the operational environment akin to that of an insider and the capability to access even wholly unconnected or only partly air-gapped industrial environments. It had a dual operational effect: physically degrading industrial equipment while clandestinely obscuring the activity from detection. But the attack on Ukraine’s power grid was a more operationally disruptive threat model that focused on coordinated penetration, remote access, credential compromise and direct manipulation of utility control equipment. Using spear-phishing campaigns, stolen credentials and remote administration tools and malware such as BlackEnergy and KillDisk, the attackers were able to infiltrate utility networks and disrupt the flow of electricity.

The aim was to disrupt service quickly, with widespread power outages and paralysis of operations at electricity companies, control centers, not long-term covert sabotage. While Stuxnet was unique in its ability to manipulate industrial processes, the attack showed how adversaries could threaten grid operations by exploiting IT-to-OT lateral movement and remote access to SCADA systems. Both show the consequences of poor network segmentation, weak authentication, poor monitoring, insecure legacy protocols, limited visibility across IT and OT environments and poor protection of operational communication channels. Moreover, both attacks highlight that relying on perimeter security is insufficient for modern smart grid defense. It demands a multi-layered security strategy, including secure authentication, intrusion detection, anomaly analysis, endpoint hardening and secure protocol design with continuous monitoring complemented by incident response plans and zero-trust architectures. Thus, the two scenarios highlight that integrated smart grid systems require strong cyber-physical defense capabilities, enabling them not only to prevent covert sabotage attacks but also to prevent large-scale operational disruptions.

### Quality of service (QoS)

**Fig. 28:**
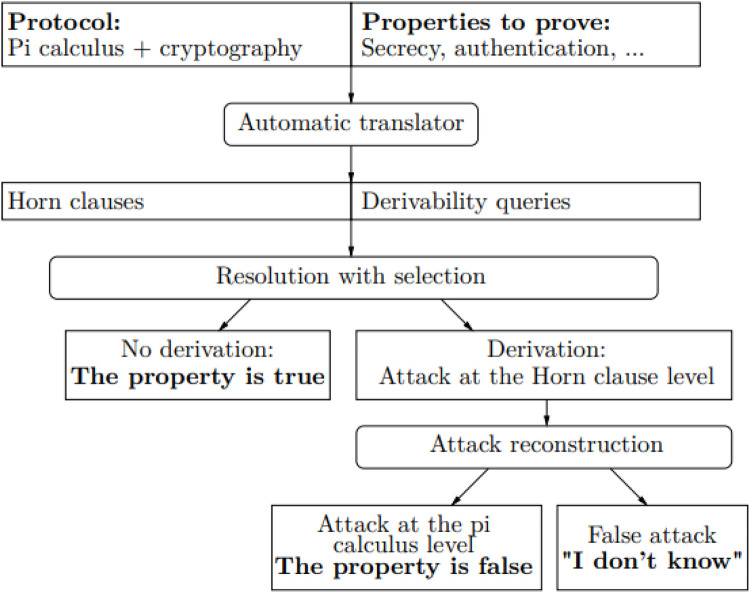
Structure of ProVerif [116].

The goal of the smart grid is to increase the dependability and efficiency of the power grids. This is achieved through the amalgamation of automated control, sensing and metering technology, cutting edge energy management strategies, and contemporary communication infrastructure. The two most important requirements for the communications network in a smart grid are security and QoS. The various indicators of service quality include outages, potential delays, and transmission error probability. In an AMI communication network, it is recommended that different degrees of QoS be provided to power users [113]. For instance, power usage report from a factory or important public department need to be given priority over a report from a home or office. While the QoS requirements for low priority power users may be relatively loosened, the AMI network should offer stronger dependability and QoS requirements in transmission for high priority power users [5,114,115]. Fig. 28 shows how to use practical aspects as a roadmap of future developments for IoT-assisted smart grids.

## Phase 5: critical evaluation of existing countermeasures

This phase summarized not only the evaluated countermeasures and protection solutions but also the rigorous testing conducted in this regard. The analysis identified practical, lightweight methods for low-cost, resource-constrained Smart Grid devices, based on theoretical procedures that would otherwise yield considerable computational or communication overhead in real-world implementations. We focus on lightweight authentication, secure session initiation, scalable key management, TLS/DTLS optimization, intrusion detection systems, and anomaly detection frameworks with a system architecture for secure SCADA communication.

### Verification methods

This sub-section describes some of the most popular tools for the verification of security protocols. These verification methods include Automated Validation of Internet Security Protocols and Applications (AVISPA), Burrows, Abadi, and Needham (BAN) logic, Real or Random (RoR) model, and Protocol Verifier (ProVerif).

• **AVISPA tool:** This is a security analysis tool that was developed in January 1st, 2003. It is utilized to assess the security of internet protocols, confirming their security status [39]. In a nutshell, AVISPA is an application tool that offers a suite of applications and modules for constructing and evaluating the security of Internet protocols and applications. Using the Dolev-Yao attacker model, it can evaluate the security elements of numerous integrated frameworks. The various services provided by this tool include formal verification, protocol analysis, automated testing and vulnerability discovery [10,117,118]. In these application domains, AVISPA exhibits low false positive rates. However, one of its limitations is that it operates under the assumption of perfect cryptography. This means that AVISPA may not be suitable for analyzing protocols involving primitives or cryptographic mechanisms outside the scope of the Dolev-Yao model. It may also be difficult for non-experts to use due to its low-level nature [119]. The general structure of AVISPA is shown in Fig. 27.
• **BAN logic:** This analysis tool first appeared in 2017 and is a widely for authentication and identity management verification. It aims to preserve privacy, maintain data integrity, prevent repudiation, and ensure traceability. The verification provided by BAN logic helps in ensuring that the security system can withstand various security attacks, such as man-in-the-middle, replay, and impersonations. Basically, the designed authentication mechanisms are evaluated by BAN logic to demonstrate that they are resilient to these threats [10]. However, the limitation of BAN is that it lacks good semantics with a clear meaning in terms of knowledge and possible universes. In addition, it is easy for the BAN logic to approve protocols which are, in practice, insecure [52].
• **RoR model:** This verification tool first appeared in 2014 and is a powerful tool for anomaly detection and fraud detection. Using this tool, it is able to distinguish between real data and random noise with high accuracy. In addition, RoR can be deployed to identify anomalies and fraudulent activity in a variety of applications [79]. The ROR model has been demonstrated to be more powerful than other security verification tools [120]. These features have posted RoR as one of the most reliable security verification tool for encryption systems, making it applicable to newer authentication protocols as well.
• **ProVerif:** Applied π-calculus forms the foundation of the security validation of ProVerif, which is the widely used formal and automated verification tool [58]. To perform unbounded verification for a class of protocols on unbounded message space, this tool leverages on an abstraction of new nonce generation. It can handle an endless number of cryptographic primitives (such as hash functions, shared and public key cryptography) and protocol sessions. In addition, ProVerif allows for the modeling of any equational theory [12]. Several cryptographic protocols and related security goals can be formally encoded using ProVerif input language, enabling automatic validation of the asserted security attributes. In this tool, it is presumed that cryptography is flawless, meaning that an attacker can only carry out cryptographic operations if they have the necessary keys. This means that it can only employ the user-specified cryptographic primitives, and cannot use any polynomial-time algorithms. ProVerif can demonstrate a number of security features, including correspondences, reachability, and observable equivalencies. These security properties are especially interesting to the security community since they make it possible to analyze privacy, authentication, and secrecy features. In the event that the desired qualities are not met, this tool can also recreate attacks [121]. This is achieved by converting the processes and adversarial activities into a series of Horn clauses, which are then utilized to automatically prove queries. Using the queries that were launched, ProVerif executes the procedures and looks for a legitimate security hole. Specifically, the secrecy property of ProVerif checks whether an attacker can decipher an encrypted term from communications sent over a public channel [86]. The Structure of ProVerif is shown in Fig. 28,

### Authentication approaches in SG

The recent past has seen the development of several security techniques to protect the smart grids. However, there is a need to develop efficient and secure solution for the smart grids based on ingenious and secure lightweight authentication techniques. These lightweight authentication systems not only address specific cyber-security concerns [122] but also help minimize energy consumption, and detecting illegitimate requests. In addition, smart grid protection requires the preservation of data confidentiality, and prevention of wrong billing. All these security features are critical for the enhancement of smart grid security [123,124]. Table 3 compares and contrasts some of the smart grid authentication techniques.

**Table 3:** Smart grid authentication methods.

Reference	Authentication approach	Mechanism	Performance	Limitation
[[125], [126], [127], [128], [129], [130], [131], [132], [133], [134], [135], [136], [137]]	Blockchain	To ensure transparency, security, and immutability, the blockchain works by recording transactions in blocks, validating them through consensus, linking the blocks cryptographically, and distributing the ledger across all network participants.	Supports secure authentication; provides secure authentication; facilitates tracking and decentralized verification; supports identity privacy protection.	High computation cost; inefficiency; scalability limits; dependency on centralized entities; neglect of privacy risks; not evaluated against Sybil attacks.
[12,39,52,138]	Hash functions	Is a mathematical algorithm that converts a digital input of any length into a fixed-size alphanumeric string called a hash value, or digest. It is a one-way function, meaning that it is computationally infeasible to reverse the digest and determine the original input from the hash value.	Prevents impersonation and repudiation attacks; low computation costs.	Collision is still possible, where two different inputs produce the same hash output; the limited input volume can only process inputs of a certain length.
[43,139]	Token	Relevant security codes issued by the Trusted Control Server (TCS) for SM and USP to register for TCP.	It has been shown to be resilient against ephemeral secret leakage, side-channel, man-in-the-middle, and impersonation attacks; requires minimal communication overhead; exhibits relatively low execution time.	Require additional resources for token generation and management, which could impact performance and scalability.
[9,76,140]	Merkle tree	A Merkle tree is a binary data structure built from a collection of leaf nodes, where every internal node represents the hash value computed from its left and right child nodes child. Basically, each internal node of a Merkle tree is a hash of its left and right children.	Enhances the speed of information authentication.	High computational cost
[12,76]	Merkle hash tree	A Merkle hash tree organizes data like a literal tree, reducing system strain for authentication and secure communication.	Minimizes computational overheads; prevents attacks such as replay and message analysis.	Cannot prevent DoS attack
[76,[141], [142], [143]]	ECC	Elliptic Curve Cryptography (ECC) is a public key cryptography method that relies on the algebraic structure of elliptic curves over finite fields.	Employs lightweight computation and energy-efficient methods to accommodate the constraints of SG devices’ low processing and power requirements.	Preloading passwords between the home area network and specific devices limits scalability and imposes the overhead of maintaining a password repository table.
[[141], [142], [143], [144], [145]]	Diffie-Hellman	Key agreement and mutual authentication for distributed SM and smart appliances present in the entire SG network. The SM along with corresponding smart appliances authenticates each other using a joint session key and hash-based authentication scheme.	Lightweigh;t strong key agreement and mutual authentication.	The computational complexity is elevated because certain operations exhibit exponential time complexity.
[76,144]	Password	Generates a new password at the beginning of each authenticated session. The generated passwords are short-lived and are automatically generated based on multiple parameters, such as local time, geographical location, and device ID.	Has the potential to provide forward and backward secrecy.	A non-trivial operation results in sophisticated and costly implementation.
[39,76,146]	Hardware	Hardware-based authentication mechanisms work by utilizing a Hardware Security Module (HSM) or a Trusted Platform Module (TPM) to secure the device’s secure storage. HSMs are regarded as among the most reliable means of secret storage; they are capable of securely storing cryptographic keys	Assures the confidentiality and integrity of the data exchanged within the network.	Costly due to the need to upgrade the devices.
[76,141,144]	Signature	The message sender generates a digital signature by applying a hash function to the message and then encrypting the hash value with their private key. The recipient of the message can then verify the authenticity of the message by decrypting the digital signature using the sender’s public key and comparing the resulting hash value with the hash value of the received message.	Mitigates memory overheads; minimizes the signature size.	The proposed scheme, despite its low computation complexity and authentication delay, does not effectively address the key agreement problem
[70,76,147,148]	Biometric	Biometric based authentication mechanisms rely on unique physical or behavioral characteristics of individuals to verify their identity. In the context of SG security, biometric authentication mechanisms can be used to provide secure and convenient access control to SG components.	Thwarts impersonation and forgery attacks; The deployed biometric data cannot be lost or forgotten.	Non-trivial in terms of its computational complexity; Such approach uses a costly gadget to collect biometric information.
[5,12,149]	Two-way authentication applied to both unicast and multicast communication scenarios	Two-way authentication is a security process where two entities involved in a communication verify each other’s identity before exchanging sensitive information. This ensures that both parties are legitimate and minimizes the risk of impersonation or unauthorized access.	Defends against attacks such as brute force, replay, DoS, and MitM attacks; low network overhead; It reduces storage and bandwidth overheads.	Does not address authentication between SM and appliances.

### Relationship between proposed security approaches and standardized cryptographic

The proposed work does not introduce a new security protocol but offers a compiled and analytical synthesis of existing authentication and key agreement procedures used in smart grids and IoT. Previous protocols detail the use of established cryptographic mechanisms, such as Transport Layer Security (TLS) (e.g., RFC 5246), Diffie–Hellman key exchange (RFC 2631) and hash-based message authentication (RFC 2104) and evaluate their suitability for constrained smart grid environments. It is worth noting that the study emphasizes how these protocols leverage well-established cryptographic primitives such as elliptic curve cryptography, hashing and symmetric operations, while also acknowledging their limits in terms of processing complexity, scalability and resistance to new attacks. This work is not a proposed cryptographic standard with new primitives; it is instead an attempt to systematically compare and contrast existing security protocols in terms of their resilience against certain well-known potential attack vectors, e.g., replay, impersonation and man-in-the-middle attacks. Table 4

**Table 4:** Comparative Analysis of Smart Grid and IoT Communication Protocols.

Protocol / Security Mechanism	Computational Complexity	Communication Overhead	Scalability	Suitability for Resource-Constrained Devices	Main Advantages	Main Limitations
HTTP	High	High	Moderate	Low	Widely supported, interoperable, easy cloud integration	Large headers, high latency, inefficient for constrained IoT devices
MQTT	Low	Low	High	High	Lightweight publish/subscribe model, efficient bandwidth usage, scalable for AMI and IoT systems	Depends on broker availability, requires additional security configuration
CoAP	Very Low	Very Low	High	Very High	Optimized for constrained devices, low power consumption, UDP-based lightweight communication	Less reliable in unstable networks, limited native congestion handling
ZigBee	Low	Low	Moderate	High	Low energy consumption, mesh networking capability, suitable for sensor networks	Limited communication range and bandwidth
6LoWPAN	Low	Very Low	High	Very High	IPv6 support for constrained devices, efficient packet compression	Security depends on upper-layer protocols
TLS / DTLS	High	Moderate to High	Moderate	Moderate	Strong encryption, authentication, secure communication channels	Handshake overhead, certificate management complexity, resource consumption
ECC-Based Authentication Schemes	Moderate	Low	High	High	Strong security with smaller key sizes, reduced energy consumption, suitable for IoT	Requires secure key management and implementation optimization
Lightweight Key Management Protocols	Low	Low	Very High	Very High	Efficient session establishment, scalable group key management, reduced communication cost	Potential synchronization and rekeying challenges in large-scale deployments

### Data collection

A systematic and adaptive methodology was applied for the literature collection and analysis for cybersecurity research for smart grids, IoT, AMI and SCADA in this work to improve methodological transparency and analytical rigor. The data collection phase was based on a huge number of scientific databases and indexing systems such as Elsevier ScienceDirect, IEEE Xplore, SpringerLink, Wiley Online Library, MDPI journals and Google Scholar.

Other technical references were obtained from RFC specifications, industrial cybersecurity reports, smart grid standards and regulatory regulations on IoT and SCADA security. The literature review was primarily limited to papers published between 2010 and 2025 to cover both fundamental research and recent advances in smart grid cybersecurity. Search and data gathering methods used dynamic keyword combinations and iterative query-refining techniques. The keywords were combinations of “smart grid cybersecurity", “SCADA attacks", “IoT authentication", “AMI security", “false data injection", "TLS/DTLS", “lightweight cryptography", “MQTT security", “CoAP security", “ransomware in smart grids", “cyber-physical attacks" and “lightweight key management". The search results were systematically improved by using Boolean operators, citation chaining and relevance ranking to enhance the inclusion of relevant studies. In the preprocessing stage, duplicate records, irrelevant publications, incomplete manuscripts, non-peer-reviewed articles and work with little technical or experimental contribution were excluded. In the preprocessing stage, titles were screened, abstracts filtered, keywords normalized, themes categorized and relevance validated against the review objectives. The studies were prioritized based on citation impact, quality of publication, technical depth, relevance to smart grid and IoT cybersecurity and contribution to communication protocols, attack models or defence mechanisms. The systematic categorization of the literature into thematic clusters is consistent with the paper's structure and covers smart grid architectures, communication protocols, authentication schemes, SCADA vulnerabilities, cyberattack classifications and lightweight cryptographic and scalable key management strategies for dynamic analytical processing. These real-time, dynamic filtering and categorization mechanisms enabled on-the-fly enhancement of the analytical methodology and comparison of existing approaches based on factors such as computational complexity, communication overheads, scalability, interoperability with current standards used in smart grid frameworks and availability/full compatibility with resource-constrained smart grid devices. Specifically, a thematic clustering method was used to systemically divide relevant literature into groups that aligned with the structure of the paper: smart grid architecture, communication protocols, authentication schemes, SCADA vulnerabilities, cyber-attack types and classes such as home area networks (HANs) and outspread areas (DIS), lightweight cryptography schemes and scalable key management policies that enabled dynamic analytical processing. To that end, this dynamic filtering and categorization process enabled continuous improvement of the analytical framework and the performance measurement and comparison of existing methodologies with respect to their computational complexity, communication overhead, scalability, interoperability and adequacy for resource-constrained smart grid devices.

### Comparative analysis of existing security mechanisms and communication technologies

In order to enhance the analytical depth, this research has examined the existing authentication schemes, key management schemes, protocol verification tools and communication technologies in terms of quantifiable performance and deployment criteria. A comparison was made using computational complexity, communication overhead, scalability, deployment complexity, energy efficiency, resilience to cyberattacks, and suitability for resource-constrained Smart Grid devices as shown in Table 5.

**Table 5:** Comparative Analysis Security Mechanisms and Communication Technologies.

Category	Computational Overhead	Scalability	Deployment Complexity	Energy Efficiency	Attack Resilience	Suitability for Resource-Constrained Devices
ECC-based Authentication	Moderate	High	Moderate	High	High	High
Certificate-based Authentication	High	Moderate	High	Low	High	Low
Centralized Key Management	Low	Moderate	Low	High	Moderate	Moderate
Hierarchical/Group Key Management	Low	High	Moderate	High	High	High
BAN Logic	Very Low	High	Low	High	Theoretical Validation	High
AVISPA	Low	High	Moderate	High	High	High
Scyther	Low	High	Moderate	High	High	High
HTTP	High	Moderate	Low	Low	Moderate	Low
MQTT	Low	High	Low	High	High	High
CoAP	Very Low	High	Low	Very High	Moderate	Very High
ZigBee	Low	Moderate	Moderate	High	Moderate	High
6LoWPAN	Low	High	Moderate	Very High	Moderate	Very High

### Research gaps

Based on the above discussions, the following are the major research gaps in this domain, which need to be explored further.

• *Maintaining privacy:* Smart networks collect and process huge data, some of which might be highly sensitive. As such, maintaining customer privacy is critical. Therefore, there is a need for the development of privacy technologies [14].
• *Communication technologies:* Data inconsistencies among the various heterogeneous communication technologies complicate security and QoS provision in IoT-assisted smart grid systems [42].
• *Limited resources in the SMs*: The smart meters are limited in terms of power, storage and communication capabilities. To address the various smart grid attacks, these devices require lightweight authentication methods that are not resource-intensive.
• *Limitations of current authentication methods:* most of the current lightweight authentication schemes deployed in smart grids do not fully address some of the attacks. As such, these smart networks are still vulnerable to attacks. There is therefore need for efficient but robust security techniques that could effectively protect the smart grids and their resource-limited devices such as smart meters.
• *Advanced data analysis techniques*: To extract valuable insights from the smart grid-generated data, there is need for enhanced and effective analysis techniques to deal with the huge amount of data generated by smart meters.
• *Cyber-attacks*: Information Communication Technologies (ICTs) play a critical role in enabling real-time monitoring, control, and analysis of smart grid network systems. However, these networks are vulnerable to numerous security threats. As cyber threats evolve and technology advances, cyber-attack threats are expected to become more elusive and sophisticated. Therefore, future research work should focus on the development of effective strategies of dealing with these complicated and elusive security threats [150].

### Comparative analysis of existing security mechanisms and communication technologies

In order to enhance the analytical depth, this research has examined the existing authentication schemes, key management schemes, protocol verification tools and communication technologies in terms of quantifiable performance and deployment criteria. A comparison was made using computational complexity, communication overhead, scalability, deployment complexity, energy efficiency, resilience to cyberattacks, and suitability for resource-constrained Smart Grid devices as the show in the Table 6,Table 7

**Table 6:** Comparative Analysis Security Mechanisms and Communication Technologies.

Category	Computational Overhead	Scalability	Deployment Complexity	Energy Efficiency	Attack Resilience	Suitability for Resource-Constrained Devices
ECC-based Authentication	Moderate	High	Moderate	High	High	High
Certificate-based Authentication	High	Moderate	High	Low	High	Low
Centralized Key Management	Low	Moderate	Low	High	Moderate	Moderate
Hierarchical/Group Key Management	Low	High	Moderate	High	High	High
BAN Logic	Very Low	High	Low	High	Theoretical Validation	High
AVISPA	Low	High	Moderate	High	High	High
Scyther	Low	High	Moderate	High	High	High
HTTP	High	Moderate	Low	Low	Moderate	Low
MQTT	Low	High	Low	High	High	High
CoAP	Very Low	High	Low	Very High	Moderate	Very High
ZigBee	Low	Moderate	Moderate	High	Moderate	High
6LoWPAN	Low	High	Moderate	Very High	Moderate	Very High

**Table 7:** Unified Threat-Countermeasure Analysis Framework for Smart Grid and IoT Environments.

Attack Type	Affected Components	Violated Security Objectives	Mitigation Approaches	Remaining Limitations
Man-in-the-Middle (MITM)	Smart meters, AMI gateways, communication channels	Confidentiality, Integrity, Authentication	Mutual authentication, TLS/DTLS, ECC-based key exchange, certificate validation	Certificate management complexity, implementation errors
Replay Attack	Smart meters, AMI servers, IoT devices	Freshness, Authentication	Nonces, timestamps, sequence numbers, challenge-response mechanisms	Clock synchronization and desynchronization issues
Denial-of-Service (DoS)	AMI networks, SCADA servers, IoT gateways	Availability	Traffic filtering, intrusion detection systems (IDS), network segmentation	Resource exhaustion in constrained devices
False Data Injection (FDI)	State estimators, control centers, PMUs	Integrity, Availability	Multi-source validation, anomaly detection, machine learning, secure state estimation	Sophisticated stealth attacks may evade detection
Desynchronization Attack	Smart meters, utility servers, key management systems	Availability, Authentication	Counter resynchronization, periodic re-authentication, sliding synchronization windows	Additional communication overhead
Ransomware Attack	SCADA servers, HMI systems, utility databases	Availability, Integrity	Network segmentation, backup systems, access control, endpoint protection	IT/OT lateral movement remains challenging
Malware Propagation	SCADA workstations, edge devices, IoT gateways	Integrity, Availability	Patch management, endpoint detection and response (EDR), secure firmware updates	Legacy systems may remain vulnerable
Insider Attack	Utility operators, privileged users, databases	Confidentiality, Integrity	Role-based access control, behavioral monitoring, multi-factor authentication	Difficult to detect legitimate misuse
Spoofing Attack	Smart meters, sensors, IoT devices	Authentication, Integrity	Device authentication, digital signatures, ECC-based authentication	Credential theft risks remain
SCADA Protocol Exploitation	PLCs, RTUs, control centers	Confidentiality, Integrity, Availability	IEC 62,351, Secure DNP3, network segmentation, zero-trust architectures	Legacy infrastructure modernization is costly

## Conclusion

This paper has studies the traditional electric power networks as well as smart grids including the challenges they face. Some of these challenges include frequent power outages, gas emissions from power plants and massive electric energy consumption in smart energy networks, which are characterized by bidirectional information transfer. The findings have indicated that security of the smart grid is an essential concept that needs to be considered in real time. It is therefore imperative to emphasize the significance of creating security measures that can guarantee the efficiency and dependability of electrical energy transmission. The primary components of conventional and smart energy networks were compared in this study. In addition, this paper addresses the most significant attacks on smart networks, including their effects and the most significant fixes that have been suggested to deal with these attacks. It has been shown that IoT technology in smart energy networks facilitates the monitoring, analysis, and operation of the entire system. Therefore, high volumes of sensitive pieces of data are exchanged by these IoT devices. In this environment, any disclosure of this information can severely compromise the privacy of the participants. To support the security and stability of the smart network connections and operations, there is need for robust but secure protocols. As a result, the most crucial smart network protocols were discussed in this paper. Normally, the smart grids analyze huge volumes of data continuously, which id collected from multiple sensors. Essentially, this increases the burden of analysis at the smart grids. Therefore, we provided elaborate discussion of cloud computing, a technology that not only helps reduce the cost of storing and processing big data but also supports real-time processing. Power grid companies can also move their entire SCADA systems to a suitable cloud to enhance data processing performance. It has also been shown that QoS plays a critical role in the effective operation and management of smart girds. This is evident in modern electricity systems that integrate advanced communication, control, and monitoring technologies to optimize electricity generation, distribution, and consumption. Therefore, enhanced QoS is key to the successful operation of smart grids as it enables efficient power management, network optimization and enhanced reliability. The study of the SCADA network system has facilitated the identification of their most significant limitations and attacks to it. For instance, it has been shown that SCADA security is a difficult problem owing to the open access network deployed in these systems.

## Ethics statements

The submitted paper represents the entire research efforts and analyses of the researchers and accurately and faithfully reflects the comprehensive coverage of the topic.

## Data availability

No data was used for the research described in the article.

## CRediT authorship contribution statement

**Maha Kadhim Kabier:** Conceptualization, Methodology, Software, Writing – original draft, Writing – review & editing. **Zaid Ameen Abduljabbar:** Conceptualization, Methodology, Software, Writing – original draft, Writing – review & editing. **Mohammed Abdulridha Hussain:** Conceptualization, Methodology, Software, Writing – original draft, Writing – review & editing. **Vincent Omollo Nyangaresi:** Writing – original draft, Writing – review & editing. **Zahraa Abdullah Ali:** Writing – original draft, Writing – review & editing. **Hamid Ali Abed AL-Asadi:** Validation, Software. **Abdulla J.Y. Aldarwish:** Validation, Software. **Ali Hasan Ali:** Validation, Software. **Ali A. Yassin:** Validation, Software.

## Declaration of competing interest

The authors declare that they have no known competing financial interests or personal relationships that could have appeared to influence the work reported in this paper.
